# Time-dependent interactions of blood platelets and cancer cells, accompanied by extramedullary hematopoiesis, lead to increased platelet activation and reactivity in a mouse orthotopic model of breast cancer – implications for pulmonary and liver metastasis

**DOI:** 10.18632/aging.102933

**Published:** 2020-03-19

**Authors:** Hassan Kassassir, Kamil Karolczak, Karolina M. Siewiera, Dagmara W. Wojkowska, Marcin Braun, Cezary W. Watala

**Affiliations:** 1 Department of Haemostatic Disorders, Faculty of Health Sciences, Medical University of Lodz, Lodz, Poland; 2 Department of Pathology, Medical University of Lodz, Lodz, Poland; 3 Postgraduate School of Molecular Medicine, Warsaw Medical University, Warsaw, Poland; 4 Department of Cytobiology and Proteomics, Medical University of Lodz, Lodz, Poland

**Keywords:** platelets, metastasis, breast cancer

## Abstract

Aging has become a significant risk factor for several diseases, including breast cancer.

Platelet activation and platelet-cancer cell aggregate fractions were found to increase with tumor progression in a mouse model of breast cancer. At advanced stages of tumor development, platelets from mice with breast cancer were hyperreactive to low agonist concentrations and hyporeactive to high ones. Platelet activation and reactivity were strongly associated with breast cancer metastasis in the lungs and extramedullary hematopoiesis in the liver. A greater fraction of platelet aggregates was observed in 4T1-injected mice at the advanced stages of breast cancer. *In vitro*, platelet activation was elevated after incubation with 4T1 cells, and thrombin-stimulated platelets formed aggregates with 4T1 cells. Neither GPIbα, nor GPIIb/IIIa blocking antibodies, were able to affect platelet-cancer cell aggregation *in vitro*.

The primed circulating platelets became more sensitive to subthreshold stimuli at advanced stages of tumor development, and the formation of platelet-cancer cell aggregates increased with cancer progression. Our findings demonstrate that the age-associated progression of breast cancer cells is connected with increased platelet functioning, and that it can be manifested by the increased number of metastases and extramedullary hematopoiesis in a time-dependent-manner.

## INTRODUCTION

It has long been acknowledged that coagulopathy is associated with the development of certain tumors; the first description of the thromboembolic indicator of malignancy was given in XIX^th^ century by Armand Trousseau [1]. Recently, it has become commonly accepted that some impairments of blood hemostasis are associated with cancer progression and are important in the promotion of cancer metastasis. Epidemiological data indicate that cardiovascular complications are the leading cause of mortality among different subgroups of oncological patients [2–4]. This suggests that blood platelets are not only involved in a migration of cancer cells to premetastatic niches: they may be also affected by tumor-derived factors in such a way that increases the risk of cardiovascular complications during cancer treatment [5]. Thus, hemostatic disturbances unequivocally constitute a pivotal part of the development of cancer and may be an interesting goal for antimetastatic therapies. Blood platelets may constitute a suitable target of pharmacological strategies undertaken to decrease platelet–tumor cell cross-talk and to reduce cellular or humoral activation of blood platelets (tumor cell-induced platelet aggregation, TCIPA) and thus, to suppress an enhanced risk of thrombotic events in cancer patients [6]. On the other hand, platelets easily interact with cancer cells in circulation, thus facilitating metastasis, a process presumably modulated by antiplatelet drugs [7, 8].

A few pharmacological pieces of evidence obtained with antiplatelet drugs in tumor models suggest that platelets play role in metastasis. Cilostazol, known to inhibit the hemostatic properties of platelets, has been proven to inhibit the metastasis of breast cancer to the lungs when used before inoculation of 4T1 cancer cells into the fat pads of mice and then after two weeks of cancer development [9]. In a melanoma model, the inhibition of the P2Y^12^ receptor with ticagrelor decreased the number of metastatic secondary tumors and improved the survival of animals injected with cancer cells [10]; however, platelet depletion did not change the rate of growth of primary melanoma [11]. The malignancy features of ovarian cancer cells, *i.e.* their invasiveness and ability to epithelial-to-mesenchymal transition (EMT), were significantly augmented by blood platelets, but decreased by treatment with acetylsalicylic acid and inhibition of the P2Y_12_ receptor [12].

The detailed molecular mechanism(s) underlying platelet activation in cancer are not fully understood. Presently, it can be suggested that cancer cells are able to activate a cysteine proteinase system, the so-called *cancer procoagulant*, which enhances some of the thrombotic features of blood platelets, such as adhesion to collagen [13] or secretion from intraplatelet granules, but does not act as a pro-aggregatory factor [14]. One of the most current theories considers platelet-derived microparticles (PMPs) as potential additional players in the interplay between the hemostatic system and cancer. Evidence exists that abundance of PMPs is elevated in numerous types of cancerous malignancies, such as skin, lung, gastric, colorectal, and breast cancers [15–18], but the mechanisms supporting the increase of circulating PMPs and their role in cancer metastasis are poorly understood. Zara and colleagues demonstrated that the PMPs obtained from platelets exposed to cancer cells were able to regulate the aggressiveness of the same cancer cells that induced their release. Interestingly, the nature of the circulating cancer cell was found to dictate its potential to provide pro-metastatic feedback: highly-aggressive cells typically induce greater PMPs release and uptake than less-aggressive cells. In addition, internalized PMPs can activate cancer cells through the phosphorylation of selected signaling proteins, including p38MAPK and myosin light chain [19]. Thus, it seems that the identification of the molecular determinants responsible not only for cancer cell-platelet interplay, but also for cancer cell–PMP interactions may play a key role in developing novel pharmacological strategies aimed at limiting of the efficacy of such prometastatic loops.

In addition, malignancies are associated with the presence of a procoagulant metabolic shift in blood plasma. For example, elevated levels of homocysteine have been observed in patients with a breast cancer [20, 21] and may be recognized as a hallmark of proaggregatory stimuli in blood platelets [22]. Thus, cancer cells and blood platelets can cross-talk indirectly through secreted molecules and share a common procoagulant environment.

Special attention should be paid to platelet receptors that can link platelets and cancer cells circulating in the blood. The question of whether platelet receptors may be responsible for their interactions with cancer cells seems to be a crucial one when considering the anti-metastatic aspects of antiplatelet therapy. A recent report indicates that the blockage of the GPIIb/IIIa receptor with its specific inhibitor eptifibatide can reduce the adhesion and migration properties of cancer cells, even highly invasive ones [23].

As described above, considerable evidence suggests that platelets play a part in the spread of cancer cells from the primary tumor to metastatic niches. Nevertheless, the time profile of platelet activation during the progression of cancer diseases is currently poorly understood. However, to choose the correct moment to begin cancer therapy, it is important to know the tumor development stage at which platelet activation occurs. In addition, to select the most appropriate antiplatelet therapy, *i.e.* one that is focused on the most potent pathway of platelet activation, a more detailed description of platelet activation and reactivity markers on blood platelets obtained at different time points of cancer development is needed.

As the risk of cancer development increases with age, aging is thought to be an important factor increasing the chance of cancer morbidity [24, 25]. This is also true in the case of breast cancer. Breast cancer, however, shows some variation depending on the time of onset. Even a simple classification of breast cancer as early-onset (occurring at pre-menopausal age) or late-onset (encountered at postmenopausal age) reveals that the former is generally an estrogen receptor-negative form that appears in higher-grade tumors, while the latter is typically estrogen receptor-positive and is typical for lower-grade forms of the disease [26–28].

It has been shown that the extent of platelet activation markedly increases with age [29]. Assuming that the incidence of cancer development also increases with age, the risk of platelet-cancer cell interplay appears very high at advanced age. However, it is poorly understood whether blood platelets are also activated by breast cancer when estrogen receptor-negative tumors develop at the premenopausal stage. To confirm such activation, platelet activation and reactivity were measured in an estrogen receptor-negative mouse model of highly metastatic breast cancer induced by 4T1 cells [30], with a relevance to premenopausal period.

A detailed evaluation was performed of the activity of markers associated with platelet activation and reactivity to physiological agonists during breast cancer metastasis. It was hypothesized that the expression of platelet activation / reactivity markers on the platelet surface would gradually increase following cancer cell inoculation into animals, reaching a peak at the final time points of cancer development. For this purpose, a mouse-based model of breast cancer was used consisting of an orthotopic injection of 4T1 cells in the fat pad, an approach used in previous studies of cancer progression, development and therapy [31]. Measurement was performed using flow cytometry, this being the best approach for measuring blood platelet activation and reactivity in a quasi-natural environment with minute volumes of available blood. In addition, *in vitro* models were used to directly test the influence of 4T1 cells on blood platelet activation.

## RESULTS

### Monitoring breast cancer metastasis to lungs during the five-week period of tumor development

**Figure 1:**
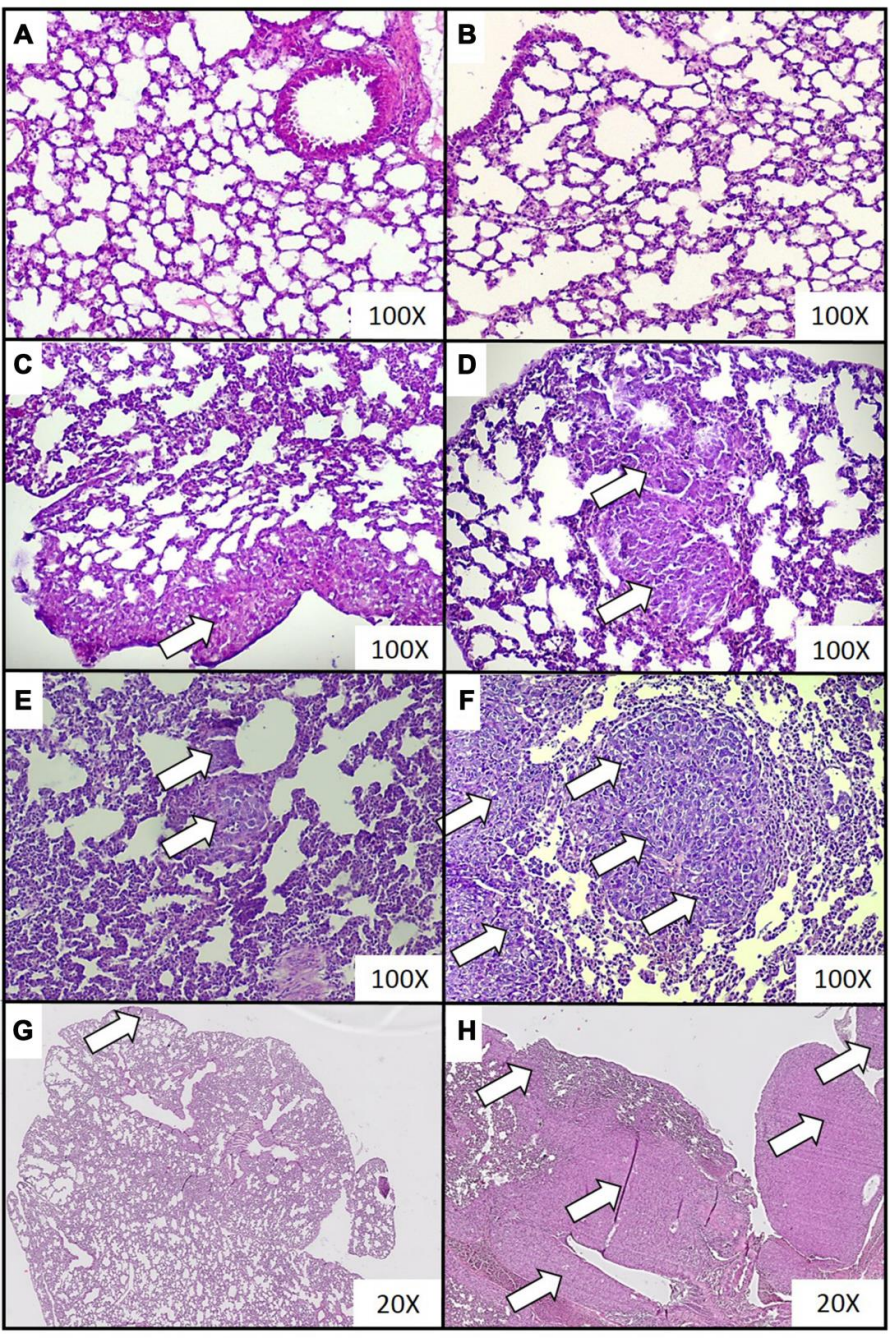
**Representative histopathological images of breast cancer metastases in lungs of mice injected with 4T1 cancer cells.** Breast cancer metastasis was investigated by histological examination of the lungs isolated from mice during necropsy carried out in animals injected with saline (control) (**A**) or after 0 (**B**), 2 (**C**), 3 (**D**), 4 (**E**) and 5 (**F**) weeks from the injection of 4T1 cancer cells. Hematoxylin and eosin staining, magnification of 100X. Additional images under lower optical magnification (20x) present breast cancer metastasis in samples of lungs resected from mice at the second (**G**) and fifth (**H**) week of breast cancer development. Cancer metastases are marked with white arrows. More experimental details are given in the Materials and methods section.

Cancer metastases were observed in higher numbers and with greater surface areas, for the largest metastases, were found in the lungs of mice sacrificed at the third, fourth and fifth week of tumor progression, compared to those sacrificed in the second week (Table 1). Representative histopathological images of the cancer metastases in lungs for different time points of disease duration are presented in Figure 1. In addition, the samples of lung tissue taken from mice presenting breast cancer at three, four and five weeks demonstrated a greater proportion of cancer metastases per surface area of analyzed histological sample and a greater number of cancer metastases per volume of the sample, than those at the first two weeks of cancer development (Table 1). Histochemical staining also revealed symptoms of inflammation; these were mainly observed at the late stages of tumor development, *i.e.* between three and five weeks (data not shown).

**Table 1:** Selected parameters of breast cancer metastasis to lungs during the five-week period of tumor development.

*Time point of breast cancer development*				
*t_o_*	*t_2_*	*t_3_*	*t_4_*	*t_5_*
Number of metastases foci *				
0; 0-0.5	1; 0-2	13; 9-19	23; 10-27	9; 8-23
Surface of the largest metastases [mm^2^] **				
0; 0- 0.02	0.05; 0-0.11	2.70; 0.48-3.30	5.40; 3.60-11.25	12.35; 2.97-24.01
Number of metastases foci/sample surface ***				
0; 0-0.1	0.01; 0-0.02	0.12; 0.07-0.24	0.17; 0.11-0.24	0.11; 0.06-0.13
Number of metastases foci/volumetric dimension of sample surface ****				
0; 0-0.1	0.01; 0-0.02	0.04; 0.02-0.06	0.05; 0.03-0.08	0.01-0.05

Results are presented as median and interquartile range (n = 4-7). Histological samples of lungs were resected immediately (two hours, t_0_) after cancer cell injection and after two (t_2_), three (t_3_), four (t_4_) and five (t_5_) weeks of cancer development. Significance of differences, estimated with Kruskal-Wallis test followed by the *post hoc* all-pairwise comparisons Conover-Inman test, were: * *P*_1,α_ < 0.001, t_5_ = t_4_ = t_3_ > t_2_ = t_0_; ** *P*_1,α_ < 0.01, t_5_ = t_4_ = t_3_ > t_2_ = t_0_; *** *P*_1,α_ < 0.01, t_5_ = t_4_ = t_3_ > t_2_ = t_0_; **** *P*_1,α_ < 0.01, t_5_ = t_4_ = t_3_ > t_2_ = t_0_.

### Estimation of extramedullary hematopoiesis in the liver and spleen during the five-week period of tumor development

**Figure 2:**
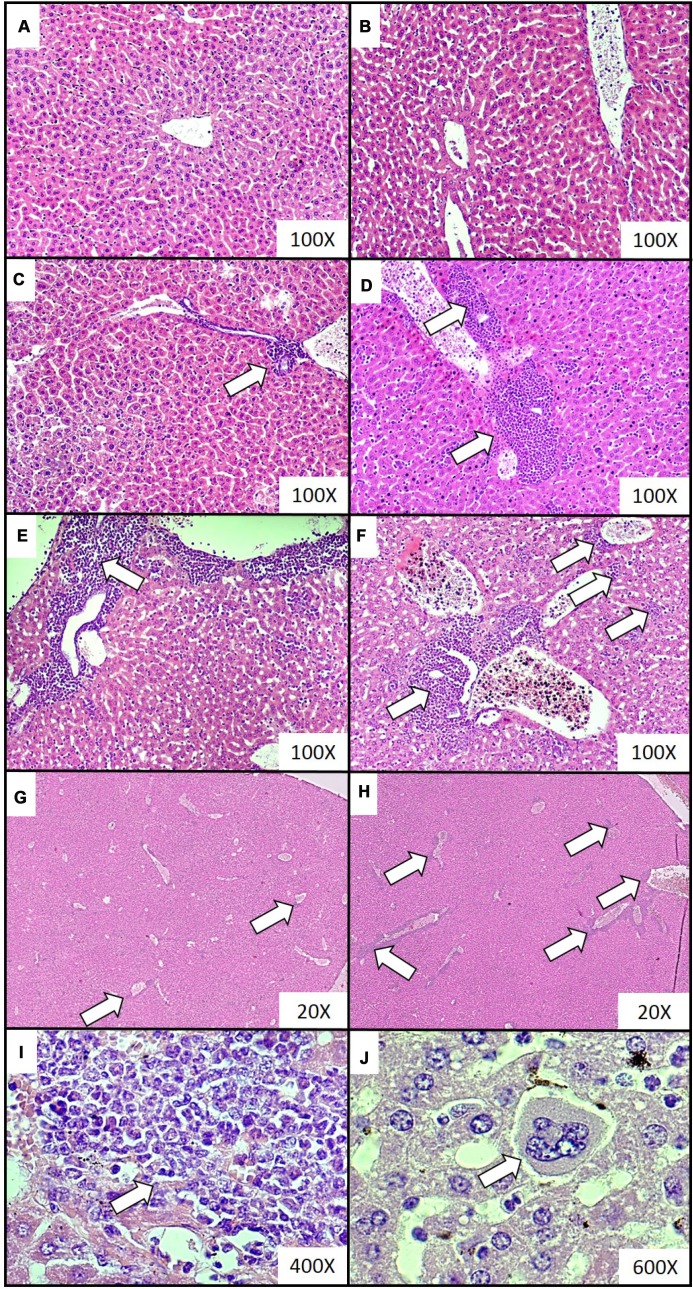
**Representative histological images of the extramedullary hematopoiesis foci in livers of mice injected with 4T1 cancer cells.** Extramedullary hematopoiesis was diagnosed histologically at increasing time intervals; observations were made based on slides from livers isolated during necropsy in animals injected with saline (control) (**A**) or after 0 (**B**), 2 (**C**), 3 (**D**), 4 (**E**) and 5 (**F**) weeks since the injection of 4T1-cancer cells. Hematoxylin and eosin staining, magnification of 100X. Additional images under lower optical magnification (20x) present extramedullary hematopoiesis foci in samples of lungs resected from mice at the second (**G**) and fifth (**H**) week of breast cancer development. The representative foci under higher magnification show the progenitor hematopoietic cells next to mature granulocytes (**I**) (400x) and a megakaryocyte (**J**) (600x). Extramedullary hematopoiesis foci are marked with white arrows. More experimental details are given in the *Materials and methods* section.

**Figure 3:**
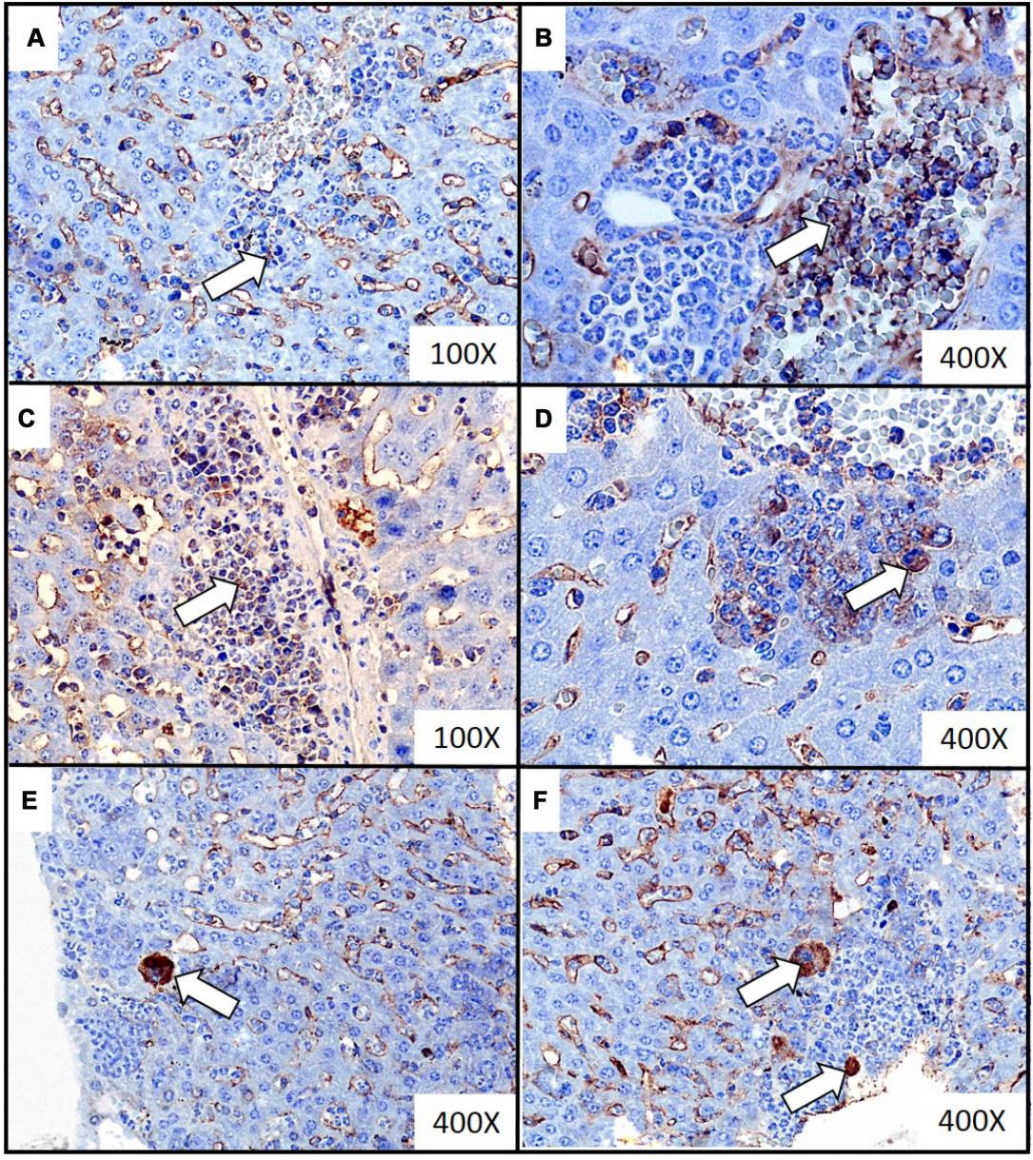
**Representative images of immunochemistry detection of the extramedullary hematopoiesis foci in liver of mice injected with 4T1 cancer cells.** Extramedullary hematopoiesis was diagnosed by immunohistochemistry staining at time interval t_5_ in slides from liver isolated during necropsy in mice injected with 4T1-cancer cells. The expressions of hematopoietic markers: CD117 (erythroid marker) (**A**, **B**), MPO (granulopoietic marker) (**C**, **D**) and FVIII (hematopoietic markers for megakaryocyte) (**E**, **F**) were detected. Additional hematoxylin staining was applied. Magnification of 100X (**A**, **C**) and 400X (**B**, **D**, **E**, **F**). Extramedullary hematopoiesis foci are marked with white arrows. More experimental details are given in the *Materials and methods* section.

**Figure 4:**
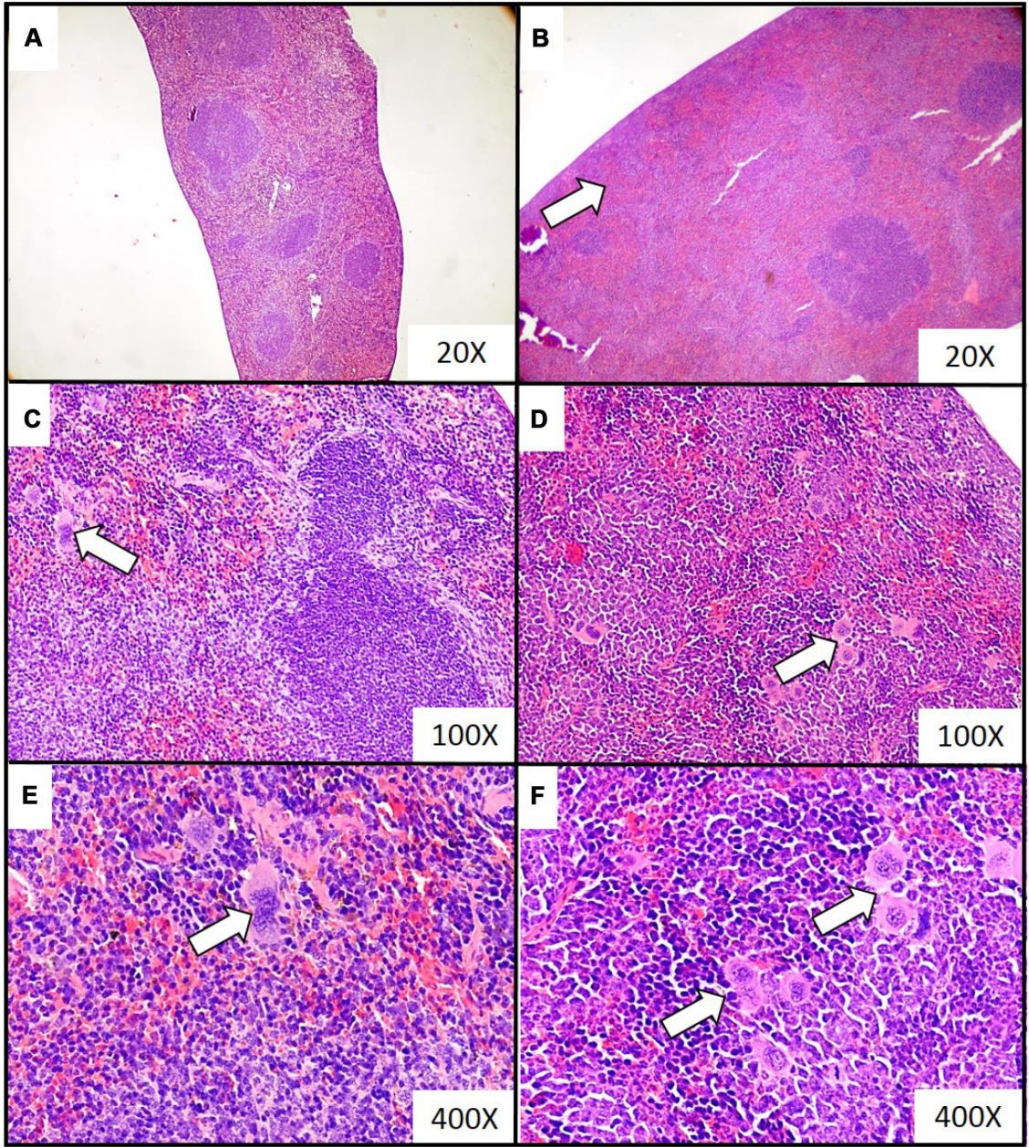
**Representative histopathological images of the extramedullary hematopoiesis foci in spleen of mice injected with 4T1 cancer cells.** Extramedullary hematopoiesis was diagnosed histologically at time interval t_5_ in slides from spleens isolated during necropsy in mice injected with saline (control) (**A**, **C**, **E**) or 4T1 cancer cells (**B**, **D**, **F**). Hematoxylin and eosin staining, magnification of 20X (**A**, **B**), 100X (**C**, **D**) and 600X (**E**, **F**). Extramedullary hematopoiesis foci are marked with white arrows. More experimental details are given in the *Materials and methods* section.

Extramedullary hematopoiesis, measured as both the number of extramedullary hematopoietic foci and the extent of extramedullary hematopoiesis, appeared to be more common in mice sacrificed at the fourth and fifth week of tumor progression, than in samples taken at two and three weeks (Table 2). Representative histological images of the extramedullary hematopoiesis foci present in the liver at different time points of disease duration are presented in Figure 2. The presence of extramedullary hematopoietic foci in the liver, detected with the use of hematoxylin and eosin staining, was further confirmed by immunohistochemistry analysis. Higher expression of FVIII (hematopoietic markers for megakaryocyte), CD117 (erythroid marker) and MPO (granulopoietic marker) was observed in the samples from mice developing breast cancer in comparison to controls (Figure 3). Hematopoietic foci were also observed in the spleens of both the mice bearing breast cancer for five weeks (4; 3-4, Me; IQR) and those injected with saline (2; 1-2, Me; IQR). This was to be expected, as the process of extramedullary hematopoiesis in spleen is known to occur throughout the lifetime of mice [32]. Representative histological images of the extramedullary hematopoietic foci identified in the spleens of mice with five-week breast cancer and of those injected with saline are presented in Figure 4.

**Table 2:** Selected parameters of extramedullary hematopoiesis in the liver during the five-week period of tumor development.

*Time point of breast cancer development*				
*t_o_*	*t_2_*	*t_3_*	*t_4_*	*t_5_*
Number of hematopoietic foci *				
0; 0-0	1; 0-3	1; 1-3	3; 2-4	3; 3-4
Extent of extramedullary hematopoiesis **				
0; 0-0	1; 0-2	2; 1-2	3; 2-4	2; 2-3

Results are presented as median and interquartile range (n = 6-8). Histological samples of liver were resected immediately (two hours, t_0_) after cancer cell injection and after two (t_2_), three (t_3_), four (t_4_) and five (t_5_) weeks of cancer development. The extramedullary hematopoiesis foci were quantified using a semiquantitative scoring method combining a 4-point score, representing the number of extramedullary hematopoietic foci (0 points: no foci and 4 points: highest number of foci) with a 4-point score, quantifying the extent of extramedullary hematopoiesis (0 point: no foci and 4 points: very high extent of extramedullary hematopoiesis). The significance of differences, estimated with the Kruskal-Wallis test followed by the *post hoc* all-pairwise comparisons Conover-Inman test, were: * *P*_1,α_ < 0.05, t_5_ = t_4_ > t_3_ > t_2_ = t_0_; ** *P*_1,α_ < 0.05, t_5_ = t_4_ > t_3_ > t_2_ = t_0._

### Changes of blood morphology during tumor progression

After four weeks, the numbers of platelets and leukocytes were found to be elevated in mice inoculated with 4T1 cells, compared to those injected with saline: (377; 337-434) *10^9^/L *vs*. (305; 296-317) *10^9^/L (*P* < 0.01) for platelets, and (59; 55-71) *10^9^/L *vs*. (0.9; 0.7-1.1) *10^9^/ (*P* < 0.001) for leukocytes; Me and IQR, significance estimated with Kruskal-Wallis test followed by the *post hoc* all-pairwise comparisons Conover-Inman test. Moreover, platelet and leukocyte counts increased with tumor progression (t_2_ *vs.* t_4_: [262; 229-316] *10^9^/L *vs*. [377; 337-434] *10^9^/L, *P* < 0.01 for platelets and [4.5; 2.1-7.7] *10^9^/L *vs*. [59; 55-71] *10^9^/L, *P* < 0.001 for leukocytes, Me and IQR). The fraction of granulocytes was also found to be greater in mice bearing breast cancer than healthy animals: 75.6; 58.7-78.3% for the 4T1-injected mice *vs*. 47.0; 39.8-49.6% for the saline-injected mice at time point t_2_ (*P* < 0.001) and 81.0; 77.6-86.8% for the 4T1-injected mice *vs*. 45.5; 43.5-49.0% for the saline-injected mice at time point t_4_ (*P* < 0.001), Me and IQR. In turn, mice with breast cancer were characterized by lower levels of lymphocytes than controls: 17.6; 14.2-33.0% for the 4T1-injected mice *vs*. 45.5; 42.8-51.6% for the saline-injected mice at t_2_ (*P* < 0.001) and 11.1; 7.0-15.0% for the 4T1-injected mice *vs*. 46.0; 43.0-48.2% for the saline-injected mice at t_4_ (*P* < 0.001), Me and IQR. No significant changes in other variables of blood morphology were observed during breast cancer development.

### Associations between blood cell counts and histological markers of lung metastases and extramedullary hematopoiesis in the liver

Negative associations were observed between the fraction of lymphocytes and the number of metastases in lungs (*R*_s_ = -0.601, *P* < 0.001), as well as with the number of extramedullary hematopoietic foci in the liver (*R*_s_ = -0.543, *P* < 0.001) of mice with breast cancer. For other blood cells, the numbers of platelets, leukocytes and the fraction of granulocytes positively correlated with the number of lung metastases (*R*_s_ = 0.637, *P* < 0.001, *R*_s_ = 0.702, *P* < 0.001, *R*_s_ = 0.581, *P* < 0.01, respectively) and the numbers of extramedullary hematopoietic outbreaks in liver (*R*_s_ = 0.568, *P* < 0.01, *R*_s_ = 0.639, *P* < 0.001, *R*_s_ = 0.529, *P* < 0.01, respectively).

### Activation of circulating blood platelets in mice at different stages of breast cancer progression

**Figure 5:**
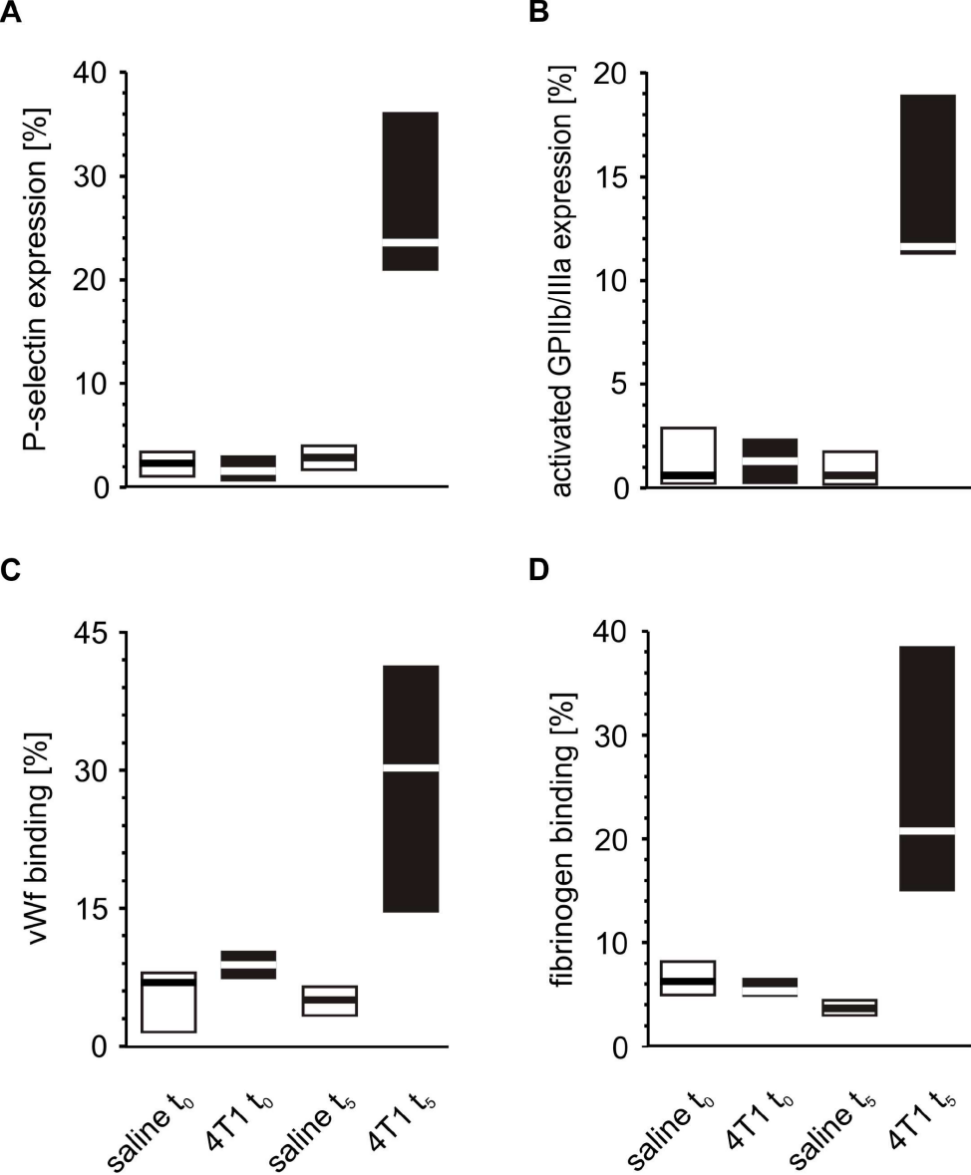
**Activation of circulating platelets in mice injected with 4T1 cancer cells or saline.** Results are presented as median (horizontal line) and interquartile range (box) (n = 8). The expressions of P-selectin (CD62P) (**A**), the active form of GPIIb/IIIa (**B**) and the binding of endogenous vWF (**C**) and endogenous fibrinogen (Fg) (**D**) on resting platelets were measured using flow cytometry in non-fixed ‘washed blood’ withdrawn immediately (t_0_) or after 5 weeks (t_5_) from the injection of mice with 4T1 cancer cells or saline. Results are expressed as the percent fraction of platelets positive for a given activation marker. More experimental details are given in the *Materials and methods* section. The statistical significance of differences, estimated with Kruskal-Wallis test followed by the *post hoc* Conover-Inman all-pairwise comparisons test, P-selectin_resting_, *P*_1,α_ < 0.001, 4T1 t_5_ > saline t_5_; *P*_1,α_ < 0.001, 4T1 t_5_ > 4T1 t_0_; active form of GPIIb/IIIa_resting_, *P*_1,α_ < 0.001, 4T1 t_5_ > saline t_5_; *P*_1,α_ < 0.001, 4T1 t_5_ > 4T1 t_0_; vWF_resting_, *P*_1,α_ < 0.001, 4T1 t_5_ > saline t_5_; *P*_1,α_ < 0.001, 4T1 t_5_ > 4T1 t_0_; Fg_resting_, *P*_1,α_ < 0.001, 4T1 t_5_ > saline t_5_; *P*_1,α_ < 0.001, 4T1 t_5_ > 4T1 t_0_.

The activation of circulating platelets was tested at time point t_0_, *i.e.* two hours after the injection of mice with 4T1 cancer cells or saline. No significant differences were found between these two groups of mice regarding any tested marker of platelet activation (Figure 5).

**Figure 6:**
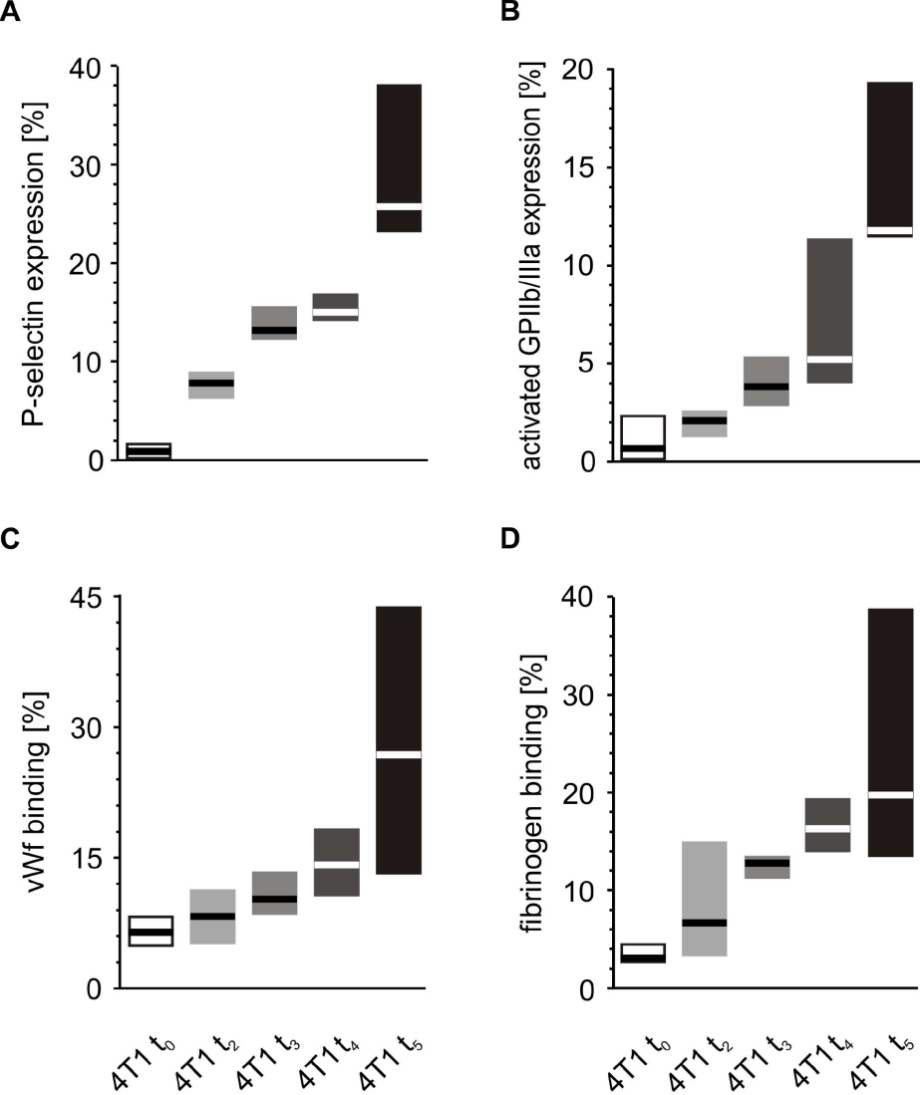
**Time-course of circulating platelet activation in mice injected with 4T1 cancer cells during 5-week breast cancer development.** Results are presented as median (horizontal line) and interquartile range (box) (n = 8). The expressions of P-selectin (CD62P) (**A**), the active form of GPIIb/IIIa (**B**) and the binding of endogenous vWF (**C**) and endogenous fibrinogen (Fg) (**D**) on resting platelets were measured using flow cytometry in non-fixed ‘washed blood’ withdraw immediately (t_0_, open boxes) or after 2 (t_2_, light grey boxes), 3 (t_3_, grey boxes), 4 (t_4_, dark grey boxes) or 5 weeks (t_5_, black boxes) from the injection of 4T1-cells. Results are expressed as the percent fraction of platelets positive for a given activation marker. More experimental details are given in the *Materials and methods* section. The statistical significance of differences, estimated with Kruskal-Wallis test followed by *post hoc* all-pairwise comparisons Conover-Inman or one-way ANOVA followed by Tukey’s multiple comparisons test, was: P-selectin_resting_, *P*_1,α_ < 0.05, 4T1 t_5_ > 4T1 t_4_ > 4T1 t_3_ > 4T1 t_2_ > 4T1 t_0_; active form of GPIIb/IIIa_resting_, *P*_1,α_ < 0.01, 4T1 t_5_ > 4T1 t_4_, 4T1 t_3_, 4T1 t_2_, 4T1 t_0;_ 4T1 t_4_ > 4T1 t_2_, 4T1 t_0_; vWF_resting_, *P*_1,α_ < 0.05, 4T1 t_5_ > 4T1 t_4_ = 4T1 t_3_ = 4T1 t_2_ > 4T1 t_0_; Fg_resting_, *P*_1,α_ < 0.01, 4T1 t_5_ > 4T1 t_2_, 4T1 t_0_; 4T1 t_4_ > 4T1 t_2_, 4T1 t_0_.

A similar comparison of the activation markers made during the fifth week after orthotopic injection revealed that tumor-bearing mice exhibited higher extents of activation in circulating resting platelets than control animals injected with saline (Figure 5). These differences observed for the markers of platelet activation progressed with time. The expression of P-selectin and the activated GPIIb/IIIa complex, as well as the binding of vWF and fibrinogen on the platelet surface increased significantly every week from t_0_ to the fifth week of tumor progression (Figure 6).

### *In vitro* reactivity to ADP or thrombin of blood platelets taken from mice injected with 4T1 cells or saline

**Figure 7:**
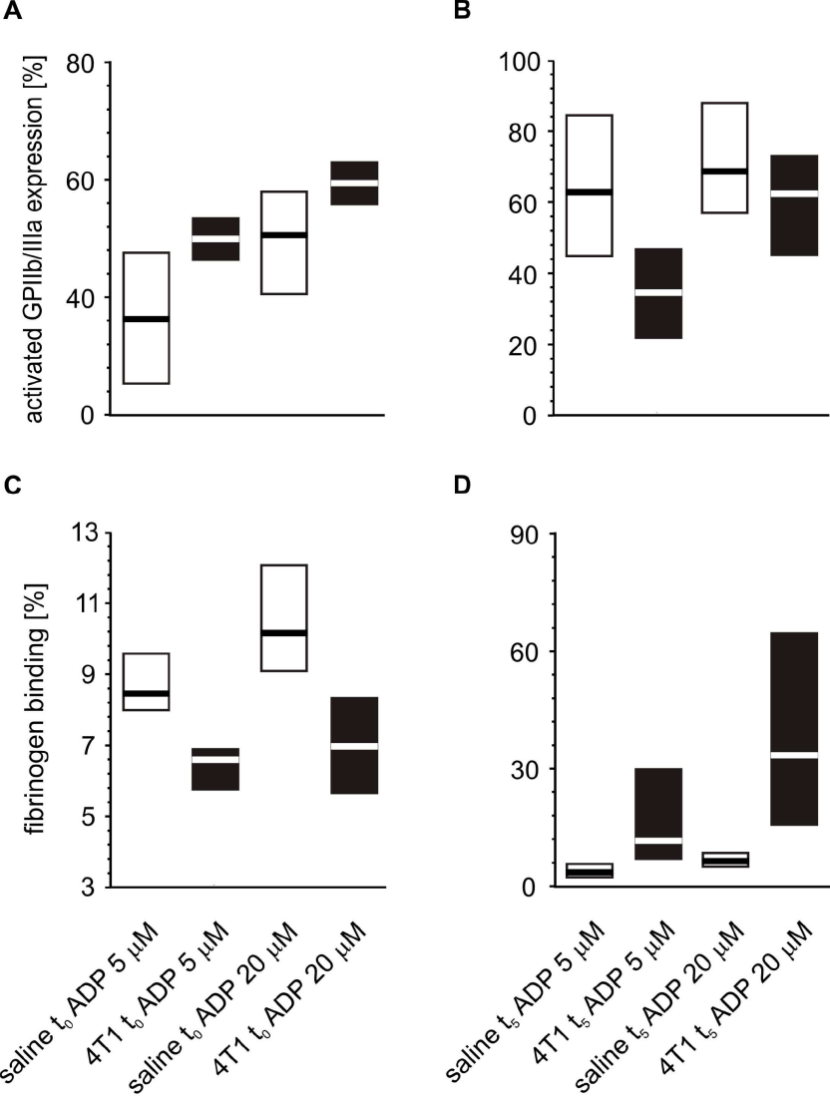
**Expressions/bindings of selected platelet surface membrane activation markers on ADP-activated blood platelets in mice injected with 4T1 cancer cells or saline.** Results are presented as median (horizontal line) and interquartile range (box) (n = 8). The expressions of the active form of GPIIb/IIIa (**A**, **B**) and binding of endogenous fibrinogen (Fg) (**C**, **D**) on platelets stimulated with ADP (5 or 20 μM) were measured using flow cytometry in non-fixed ‘washed blood’ withdraw immediately (t_0_) (**A**, **C**) or after 5 weeks (t_5_) (**B**, **D**) from the injection of 4T1-cells or saline. Results are expressed as the percent fraction of platelets positive for a given activation marker. More experimental details are given in the *Materials and methods* section. The statistical significance of differences, estimated with Kruskal-Wallis test followed by *post hoc* Conover-Inman all-pairwise comparisons or two-way ANOVA followed by Tukey’s multiple comparisons test, was: active form of GPIIb/IIIa_ADP5μM_, *P*_1,α_ < 0.01, 4T1 t_0_ > saline t_0_; *P*_1,α_ < 0.01, 4T1 t_5_ < saline t_5_; active form of GPIIb/IIIa_ADP20μM_, *P*_1,α_ < 0.01, 4T1 t_0_ > saline t_0_; *P*_1,α_ < 0.01, 4T1 t_5_ < saline t_5_; Fg_ADP5μM_, *P*_1,α_ < 0.01, 4T1 t_0_ < saline t_0_; *P*_1,α_ < 0.01, 4T1 t_5_ < saline t_5_; Fg_ADP20μM_, *P*_1,α_ < 0.01, 4T1 t_0_ < saline t_0_; *P*_1,α_ < 0.01, 4T1 t_5_ < saline t_5_.

**Figure 8:**
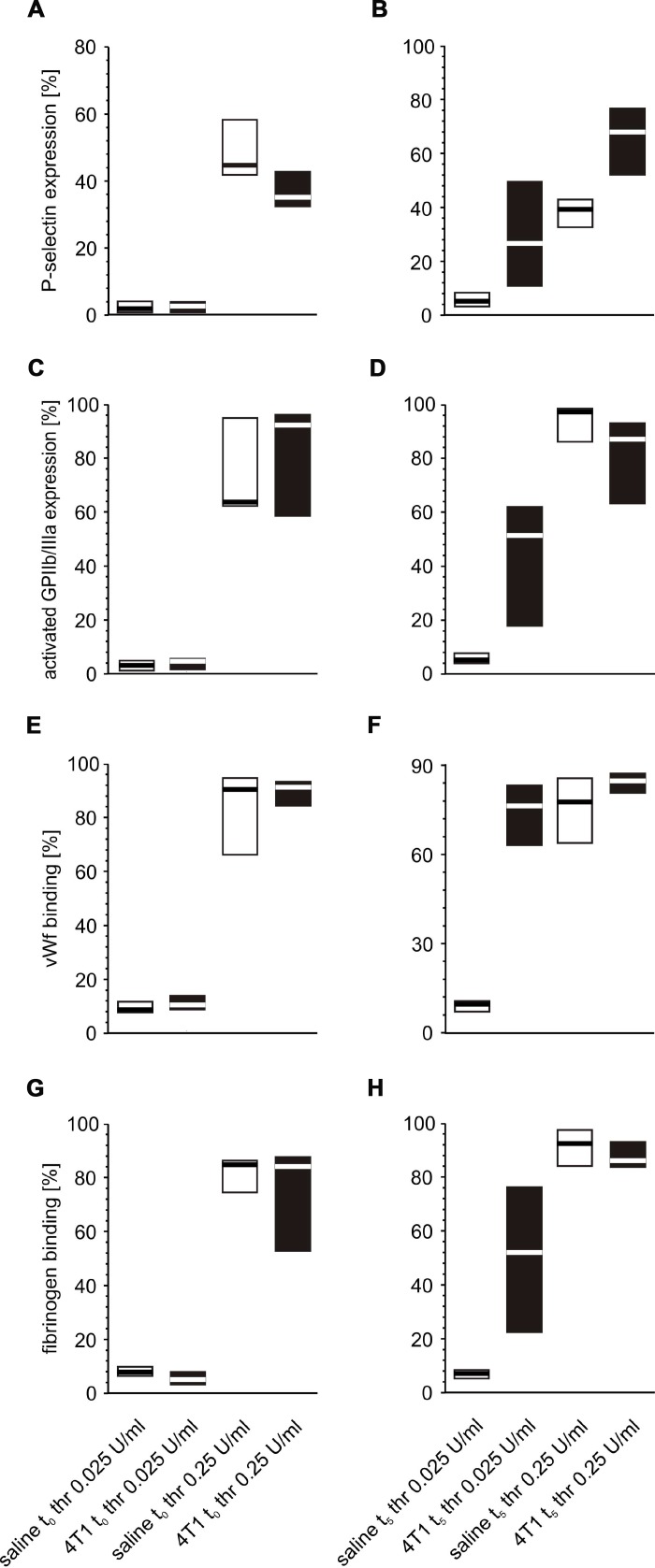
**Expression/binding of selected platelet surface membrane activation markers on thrombin-activated blood platelets in mice injected with 4T1 cancer cells or saline.** Results are presented as median (horizontal line) and interquartile range (box) (n = 8). The expressions of P-selectin (CD62P) (**A**, **B**), the active form of GPIIb/IIIa (**C**, **D**) and binding of endogenous vWF (**E**, **F**) and endogenous fibrinogen (Fg) (**G**, **H**) on platelets stimulated with thrombin (0.025 or 0.25 U/ml) were measured using flow cytometry in non-fixed ‘washed blood’ withdrawn immediately (t_0,_ **A**, **C**, **E**, **G**) or after 5 weeks (t_5_, **B**, **D**, **F**, **H**) from the injection of 4T1 cells or saline. Results are expressed as the percent fraction of platelets positive for a given activation marker. More experimental details are given in the *Materials and methods* section. The statistical significance of differences, estimated with Kruskal-Wallis test followed by *post hoc* all-pairwise comparisons Conover-Inman or two-way ANOVA followed by Tukey’s multiple comparisons test, was: P-selectin _thr0.025U/ml_, *P*_1,α_ < 0.05, 4T1 t_0_ < saline t_0_; *P*_1,α_ < 0.01, 4T1 t_5_ > saline t_5_; active form of GPIIb/IIIa _thr0.025U/ml_, *P*_1,α_ < 0.01, 4T1 t_5_ > saline t_5_; vWF _thr0.025U/ml_, *P*_1,α_ < 0.05, 4T1 t_0_ > saline t_0_; *P*_1,α_ < 0.01, 4T1 t_5_ > saline t_5_; vWF _thr0.25U/ml_, *P*_1,α_ < 0.05, 4T1 t_0_ > saline t_0_; Fg_thr0.025U/ml_, *P*_1,α_ < 0.05, 4T1 t_0_ < saline t_0_; *P*_1,α_ < 0.01, 4T1 t_5_ > saline t_5_.

Following *in vitro* platelet stimulation with ADP, the platelets of mice inoculated with 4T1 cancer cells demonstrated increased activated GPIIb/IIIa complex expression and reduced fibrinogen binding at time point t_0_ (Figure 7) than those obtained from mice injected with saline. However, the opposite situation was observed five weeks after inoculation, *i.e.* the expression of GPIIb/IIIa was reduced and the amount of bound fibrinogen on the platelet surface was increased in 4T1 mice in response to ADP stimulation (Figure 7). For thrombin-stimulated platelets, differences in platelet reactivity between 4T1- and saline-injected mice were observed only at t_5_ (Figure 8).

### Changes in the *in vitro* reactivity of blood platelets in mice during five weeks of tumor development

**Figure 9:**
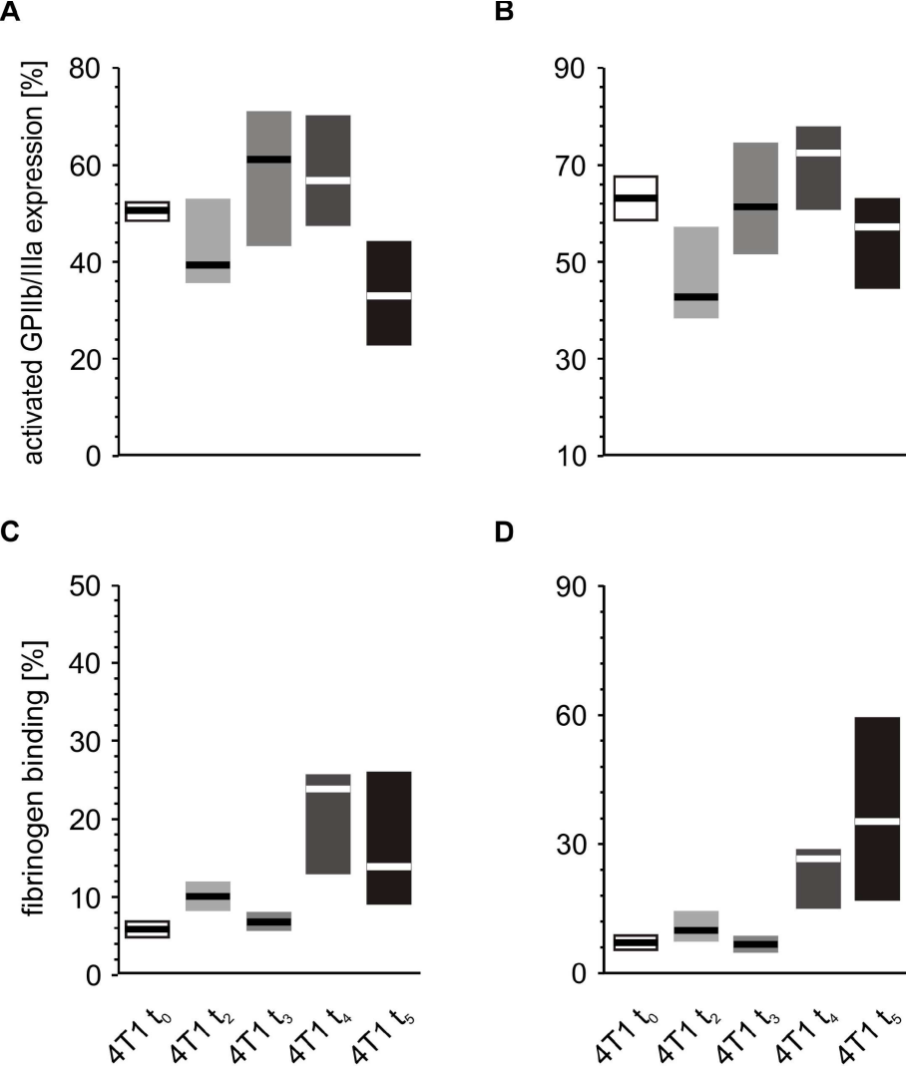
***In vitro* response to increasing concentrations of ADP of whole blood platelets from mice with orthotopic model of breast cancer.** Results are presented as median (horizontal line) and interquartile range (box) (n = 8). The expressions of the active form of GPIIb/IIIa (**A**, **B**) and the binding of endogenous fibrinogen (Fg) (**C**, **D**) on platelets stimulated with 5 μM ADP (**A**, **C**) and 20 μM ADP (**B**, **D**) were measured using flow cytometry in non-fixed ‘washed blood’ withdrawn immediately (t_0_) or after 2 (t_2_), 3 (t_3_), 4 (t_4_) or 5 weeks (t_5_) from the injection of 4T1 cells. Results are expressed as the percentage fraction of a given activation marker-positive platelets. More experimental details are given in the *Materials and methods* section. Statistical significance of differences, estimated with Kruskal-Wallis test followed by *post hoc* all-pairwise comparisons Conover-Inman or one-way ANOVA followed by Tukey’s multiple comparisons test, was: active form of GPIIb/IIIa_ADP5μM_,; *P*_1,α_ < 0.05, 4T1 t_5_ < 4T1 t_4_ = 4T1 t_3_ > 4T1 t_2_ = 4T1 t_0_; active form of GPIIb/IIIa_ADP20μM_, *P*_1,α_ < 0.01, 4T1 t_4_ > 4T1 t_2_; Fg_ADP5μM_, *P*_1,α_ < 0.05, 4T1 t_5_ = 4T1 t_4_ > 4T1 t_3_ = 4T1 t_2_ = 4T1 t_0_; Fg_ADP20μM,_ *P*_1,α_ < 0.05, 4T1 t_5_ = 4T1 t_4_ > 4T1 t_3_ < 4T1 t_2_ > 4T1 t_0._

Some fluctuations in the expression of activated GPIIb/IIIa complex on platelets stimulated with ADP were observed during the five-week course of breast cancer (Figure 9A, 9B). This expression appeared to be the highest after weeks 3 and 4, while it reached the lowest values after two and five weeks. In contrast, a significant rise in the levels of fibrinogen binding to platelets following ADP administration was observed after weeks 4 and 5.

**Figure 10:**
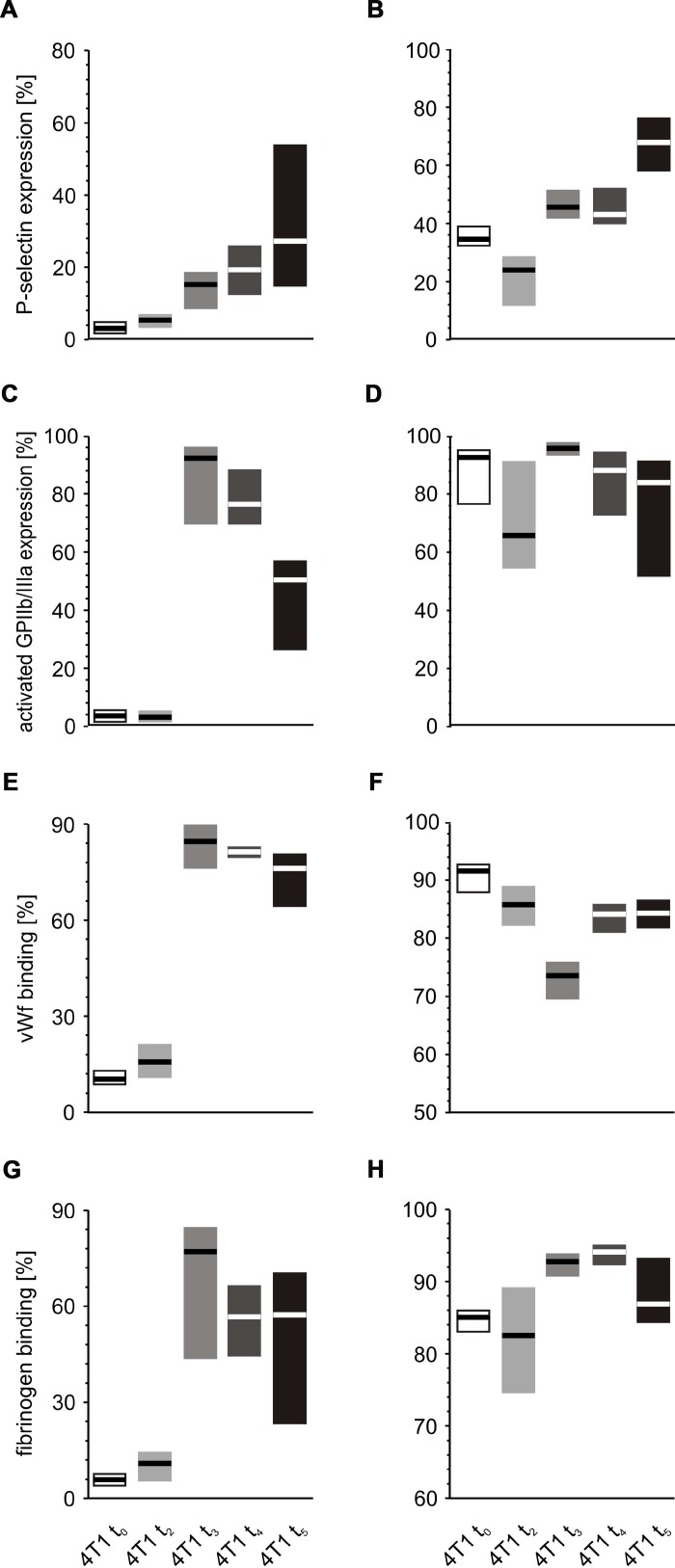
***In vitro* response of whole blood platelets from mice with orthotopic model of breast cancer to increasing concentrations of thrombin.** Results are presented as median (horizontal line) and interquartile range (box) (n = 8). The expressions of P-selectin (CD62P) (**A**), the active form of GPIIb/IIIa (**B**) and binding of endogenous vWF (**C**) and endogenous fibrinogen (Fg) (**D**) on platelets stimulated with low thrombin (0.025 U/ml) (**A**, **C**, **E**, **G**) or high thrombin (0.25 U/ml) (**B**, **D**, **F**, **H**) were measured using flow cytometry in non-fixed ‘washed blood’ withdrawn immediately (t_0_) or after 2 (t_2_), 3 (t_3_), 4 (t_4_) or 5 weeks (t_5_) from the injection of 4T1 cells. Results are expressed as the percent fraction of platelets positive for a given activation marker. More experimental details are given in the *Materials and methods* section. The statistical significance of differences, estimated with Kruskal-Wallis test followed by the *post hoc* Conover-Inman all-pairwise comparisons or one-way ANOVA followed by Tukey’s multiple comparisons test, was: P-selectin _thr0.025U/ml_, *P*_1,α_ < 0.05, 4T1 t_5_ = 4T1 t_4_ > 4T1 t_3_ > 4T1 t_2_ > 4T1 t_0_; P-selectin _thr0.25U/ml_, *P*_1,α_ < 0.001, 4T1 t_5_ > 4T1 t_4_ = 4T1 t_3_ > 4T1 t_2_ < 4T1 t_0_; active form of GPIIb/IIIa _thr0.025U/_ml, *P*1_,α_ < 0.05, 4T1 t_5_ < 4T1 t_4_ = 4T1 t_3_ > 4T1 t_2_ = 4T1 t_0_; vWF _thr0.025U/ml_, *P*_1,α_ < 0.001, 4T1 t_5_ = 4T1 t_4_ = 4T1 t_3_ > 4T1 t_2_ = 4T1 t_0_; vWF _thr0.25U/ml_, *P*_1,α_ < 0.01, 4T1 t_5_ = 4T1 t_4_ > 4T1 t_3_ < 4T1 t_2_ = 4T1 t_0_; Fg_thr0.025U/ml,_ *P*_1,α_ < 0.001, 4T1 t_5_ = 4T1 t_4_ = 4T1 t_3_ > 4T1 t_2_ = 4T1 t_0_; Fg_thr0.25U/ml_, *P*_1,α_ < 0.05, 4T1 t_5_ < 4T1 t_4_ = 4T1 t_3_ > 4T1 t_2_ = 4T1 t_0_.

Thrombin stimulation of platelets was found to be associated with increased expression of P-selectin at almost every time point during the course of the disease, peaking after the fifth week (Figure 10A, 10B). Interestingly, the expression of activated GPIIb/IIIa complex (Figure 10C, 10D) and binding of fibrinogen (Figure 10G, 10H) were found to be highest at time points t_3_ and t_4_, regardless of the thrombin concentrations. In the case of vWF, the highest binding of this protein to the platelet surface was observed after weeks 3 and 4; however, this was noted only for the lower concentration of thrombin (Figure 10E). For higher doses of thrombin, the opposite tendency occurred: the lowest values of bound vWF were noted at time points t_3_ and t_4_ (Figure 10F).

A separate series of experiments included an additional control: the injection of breast epithelium non-cancer cells (EpH4-Ev). These injections were administered at time points t_0_ and t_2_ to verify whether the possible changes in platelet reactivity observed after 4T1 injection might be due simply to the procedure of cell injection. Only these first two time points were chosen because the inoculated non-cancer cells should become neutralized by the immune system and would not be present in bloodstream after a longer time. No significant changes of platelet activation or reactivity were found for mice inoculated with EpH4-Ev cells, compared to mice injected with saline. However, two weeks after the injection with 4T1 breast cancer cells, the mice demonstrated increased activation of circulating platelets (*P* < 0.01 for the expression of activated GPIIb/IIIa complex and the binding of vWF and Fg) and elevated response to 20 μM ADP (*P* < 0.05 for CD62P and *P* < 0.01 for vWF and Fg) and 0.25 U/ml thrombin (*P* < 0.01 for CD62P and vWF) than those administered EpH4-Ev breast epithelium non-cancer cells (data not shown). In the mice inoculated with cancer cells, breast tumors were found to form two weeks after the injection of 4T1 cells. Therefore, we correctly deduced that these cells can be detected at the time point t_2_ in the bloodstream.

### Canonical correlations between the set of parameters of blood platelet activation and reactivity and the set of histological markers of lung metastases and extramedullary hematopoiesis in liver

Overall, the tested variables associated with the activation of circulating platelets in the 4T1-injected mice were found to be strongly associated with the markers of breast cancer metastasis in the lungs and those of extramedullary hematopoiesis in the liver (Table 3). In addition, the *in vitro* study revealed a significant relationship between the measured variables describing blood platelet reactivity, *i.e.* the expressions of the examined hallmarks of platelet reactivity for different concentrations of agonists, with the number of breast cancer metastases in lungs, as well as with the extramedullary hematopoiesis foci in liver (Table 3). The outcomes in Table 3 clearly demonstrate that P-selectin expression, as well as the binding of fibrinogen and vWF to resting circulating platelets or platelets agonized with thrombin are the most significant contributors explaining the extent of variability of breast cancer metastases in lungs. However, although the number of extramedullary hematopoietic foci in the liver was mostly explained by fibrinogen binding to resting, ADP-activated and low thrombin-activated platelets, it was also associated with P-selectin and GPIIb/IIIa expression and vWF binding in low thrombin-stimulated platelets.

**Table 3:** Canonical correlations between the set of blood platelet activation and reactivity parameters and the sets of histochemical markers of lung metastases and extramedullary hematopoiesis.

***metastases (set 1)***	***extracted variance [%]***	***total redundancy [%]***	***explanatory variables (set 2)***	***extracted variance [%]***	***total redundancy [%]***	**canonical correlation**	**canonical determination [R^2^]**	**P**	**Wilks' lambda**	**best contributors**
*metastases in lungs*	100.0	78.5	*plt activation and reactivity*	40.4	30.2	0.965	0.932	0.001	0.002	P-selectin_resting_, Fg_resting_, vWf_resting_, Fg_ADP20_
*metastases in lungs*	100.0	44.1	*plt activation*	100.0	42.9	0.880	0.775	0.000	0.111	P-selectin_resting_, vWf_resting_, Fg_resting_
*metastases in lungs*	100.0	57.4	*reactivity low conc. agonists*	84.0	25.4	0.837	0.701	0.001	0.091	GPIIb/IIIa^thr0.025^, vWf_thr0.025_, Fg_thr0.025_
*metastases in lungs*	100.0	34.7	*reactivity high conc. agonists*	70.6	26.3	0.720	0.518	0.015	0.158	P-selectin_thr0.25_, Fg_thr0.25_
*hematopoiesis in liver*	100.0	81.2	*plt activation and reactivity*	26.2	21.3	0.920	0.847	0.007	0.051	Fg_resting_, Fg_ADP20_, Fg_thr0.025_, vWf_thr0.025_, GPIIb/IIIa_thr0.025_, P-selectin_thr 0.025_
*hematopoiesis in liver*	100.0	42.9	*plt activation*	59.5	25	0.696	0.485	0.008	0.429	P-selectin_resting_, GPIIb/IIIa_rest_
*hematopoiesis in liver*	100.0	52.1	*reactivity low conc. agonists*	59.1	27.6	0.756	0.572	0.028	0.376	vWf_thr0.025_, Fg_thr0.025_, GPIIb/IIIa_thr0.025_, P-selectin_thr0.025_
*hematopoiesis in liver*	100.0	40.3	*reactivity high conc. agonists*	36.0	12.4	0.655	0.430	0.097	0.452	Fg_ADP20_, Fg_thr0.25_, P-selectin_thr0.25_

Rho² is a squared canonical correlation coefficient (canonical determination), which is relevant to variance between canonical variables. Total redundancy is relevant to the variance between the canonical variable for set 1 and the variables of set 2; it shows the representation of a given canonical variable by the set 2 of explanatory variables. Extracted variance is a variance between a given canonical variable and the variables that build it up; it reflects how well a given canonical variable represents a given set of variables. The Wilks’ lambda is the parameter reflecting the contribution of a set 2 to the explaining of the variance of a set 1; the lower is the Wilks’ lambda, the higher is the contribution.

### Detection of complexes of blood platelets and cancer cells in mice in the course of orthotopically-induced breast cancer

**Figure 11:**
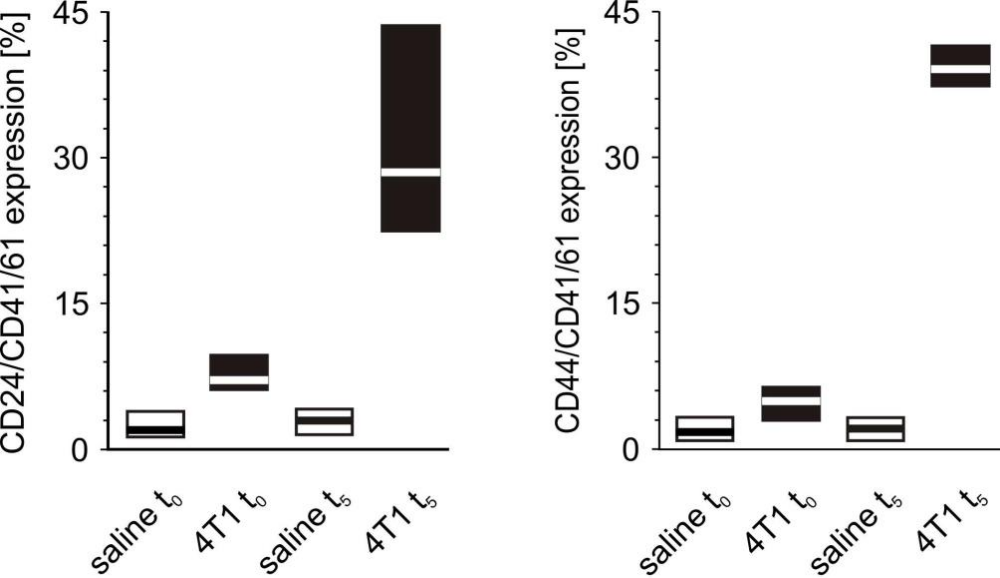
**The formation of platelet-cancer cells aggregates in mice injected with 4T1 cells or saline.** Results are presented as median (horizontal line) and interquartile range (box) (n = 8). The expressions of CD24 or CD44 within the population of the CD41/61-gated cells (platelets) were measured using flow cytometry in non-fixed ‘washed blood’ drawn immediately (t_0_) or after 5 weeks (t_5_) from the injection of 4T1 cells (n = 8). Results are expressed as the percent fraction of CD24/CD41/61 or CD44/CD41/61-positive cells. For further experimental details – see *Materials and methods* section. The statistical significance of differences, estimated with Kruskal-Wallis test followed by *post hoc* Conover-Inman all-pairwise comparisons or one-way ANOVA followed by Tukey’s multiple comparisons test, was: CD24/CD41/61-positive cells (4T1-platelet aggregates): *P*_1,α_ < 0.001, 4T1 t_0_ > saline t_0_, 4T1 t_5_ > saline t_5_; CD44/CD41/61-positive cells (4T1-platelet aggregates): *P*_1,α_ < 0.01, 4T1 t_0_ > saline t_0_; *P*_1,α_ < 0.01, 4T1 t_5_ > saline t_5_.

**Figure 12:**
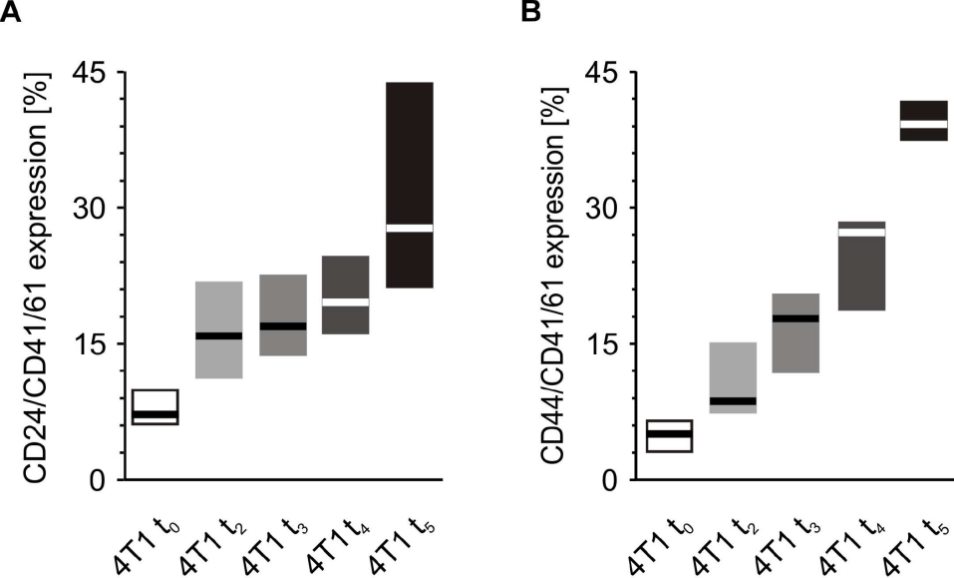
**Time-course of the formation of platelet-cancer cell aggregates during 5-week tumor progression in the mouse model of breast cancer.** Results are presented as median (horizontal line) and interquartile range (box) (n = 8). The expressions of CD24 (**A**) or CD44 (**B**) within the population of the CD41/61-gated cells (platelets) were measured using flow cytometry in non-fixed ‘washed blood’ withdrawn immediately (t_0_) or after 2 (t_2_), 3 (t_3_), 4 (t_4_) or 5 weeks (t_5_) from the injection of 4T1 cells. Results are expressed as the percent fraction of CD24/CD41/61 or CD44/CD41/61-positive cells. More experimental details are given in the *Materials and methods* section. Statistical significance of differences, estimated with Kruskal-Wallis test followed by *post hoc* Conover-Inman all-pairwise comparisons or one-way ANOVA followed by Tukey’s multiple comparisons test, was: CD24/CD41/61-positive cells (4T1-platelet aggregates), *P*_1,α_ < 0.001, 4T1 t_5_ > 4T1 t_0_; *P*_1,α_ < 0.01, 4T1 t_5_ > 4T1 t_3_, 4T1 t_5_ > 4T1 t_2_; *P*_1,α_ < 0.05, 4T1 t_5_ > 4T1 t_4_, 4T1 t_4_ > 4T1 t_0_; CD44/CD41/61-positive cells (4T1-platelet aggregates), *P*_1,α_ < 0.01, 4T1 t_5_ > 4T1 t_4_ > 4T1 t_3_ > 4T1 t_0_; *P*_1,α_ < 0.01, 4T1 t_5_ > 4T1 t_4_ > 4T1 t_2_ > 4T1 t_0_.

**Figure 13:**
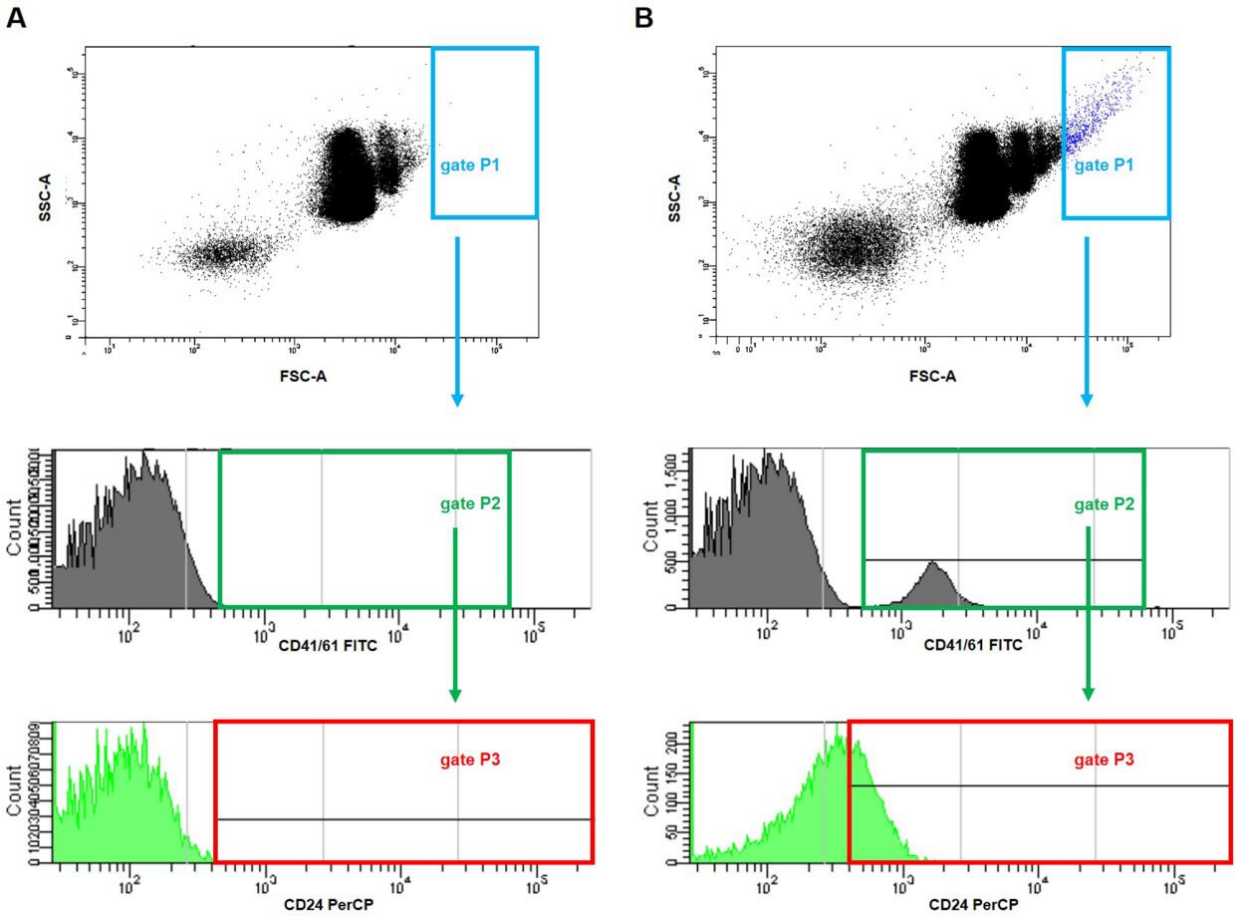
**Representative dot plots and histograms showing gating strategy for the detection of platelet-cancer cells aggregates in mice injected with 4T1 cells or saline.** The parent gate was set to the cancer cells based on FSC/SSC physical parameters (gate P1, in blue). Next, within this gated population, platelet-cancer cells aggregates were identified according to the surface presence of both CD41/61 (a unique antigen for platelets) (gate P2, in green) and CD24 (marker for cancer cells) (gate P3, corrected on the isotype binding, in red) in blood samples taken from mice five weeks after the injection with saline (**A**) or 4T1 breast cancer cells (**B**).

Before precise measurements were taken, it was verified whether CD41/61 is present on the 4T1 cells. There was no expression of this antigen on the surface of cultured mouse breast cancer 4T1 cells (data not shown). The number of CD24/CD41/61-negative and CD44/CD41/61-positive cell aggregates measured at time point t_0_ was found to be higher in mice injected with 4T1 cells in comparison to those injected with saline (Figure 11). Likewise, the number of CD24/CD41/61- and CD44/CD41/61-positive cell complexes found after five weeks was significantly higher in mice injected with 4T1 cells than in to those injected with saline (Figure 11). Furthermore, a statistically significant increase in the number of CD24/CD41/61-positive complexes was found, when blood samples taken at the fifth week of disease were compared to those taken earlier, *i.e.* at time point t_0_ and after two weeks, three weeks and four weeks of disease progression (Figure 12). Also, the numbers of CD44/CD41/61-positive aggregates steadily increased over the course of tumor progression (Figure 12): significant changes were observed between subsequent time points, apart from between the second and the third weeks. Example dot plots and histograms showing the gating strategy for detection of platelet-cancer cell aggregates are presented in Figure 13.

### Impact of 4T1 cancer cells on platelet activation *in vitro*

**Figure 14:**
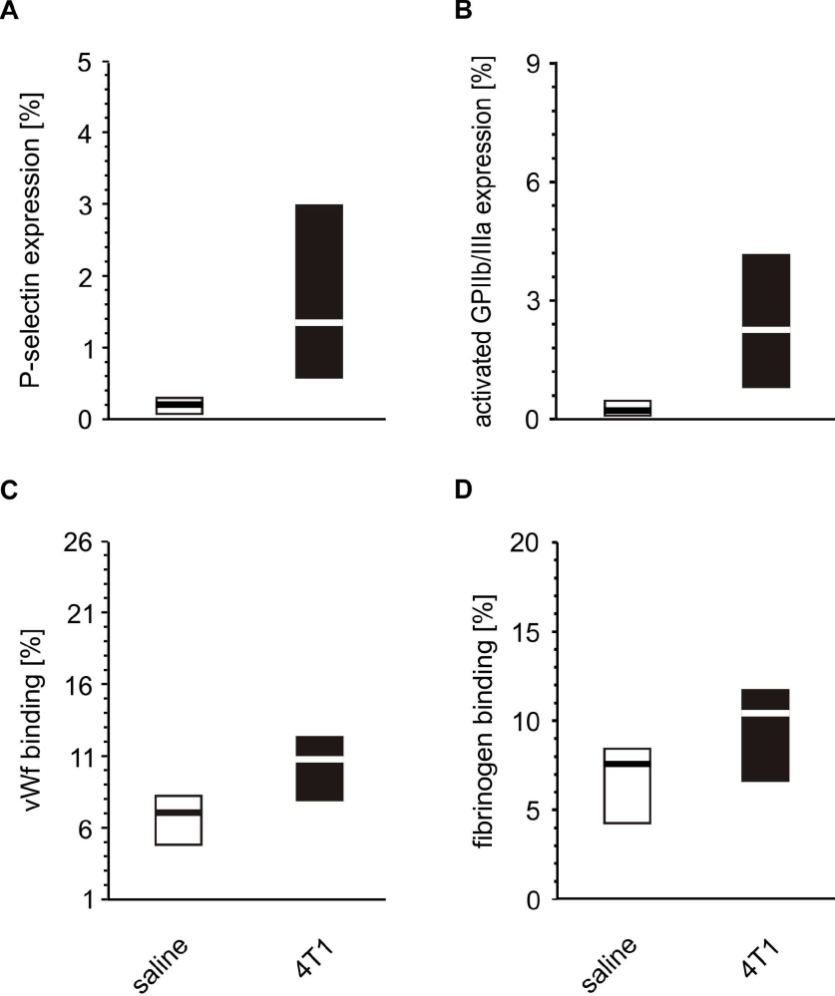
***In vitro* activation of whole blood platelets incubated with 4T1 cells.** Results are presented as median (horizontal line) and interquartile range (box) (n = 8). The expressions of P-selectin (CD62P) (**A**), the active form of GPIIb/IIIa complex (**B**) and the binding of endogenous vWF (**C**) and endogenous fibrinogen (Fg) (**D**) on platelets was measured using flow cytometry in non-fixed ‘washed blood’ incubated with 4T1-cancer cells or saline. Results are expressed as the percent fraction of the CD41/61-positive platelets. More experimental details are given in the *Materials and methods* section. The statistical significance of differences, estimated with the Mann-Whitney *U*-test, was: P-selectin, *P*_1,α_ < 0.001, 4T1 >saline; active form of GPIIb/IIIa, *P*_1,α_ < 0.001, 4T1 > saline; vWF, *P*_1,α_ < 0.01, 4T1 > saline; Fg, *P*_1,α_ < 0.05, 4T1 > saline.

Increased activation of resting platelets was observed, as evidenced by the enhanced expression of P-selectin, GPIIb/IIIa and increased binding of vWF and fibrinogen following the *in vitro* incubation of platelets with 4T1 cells, compared to those incubated with saline (Figure 14).

### *In vitro* effects of GPIbα and GPIIb/IIIa inhibitors on platelet-cancer cells aggregation

**Figure 15:**
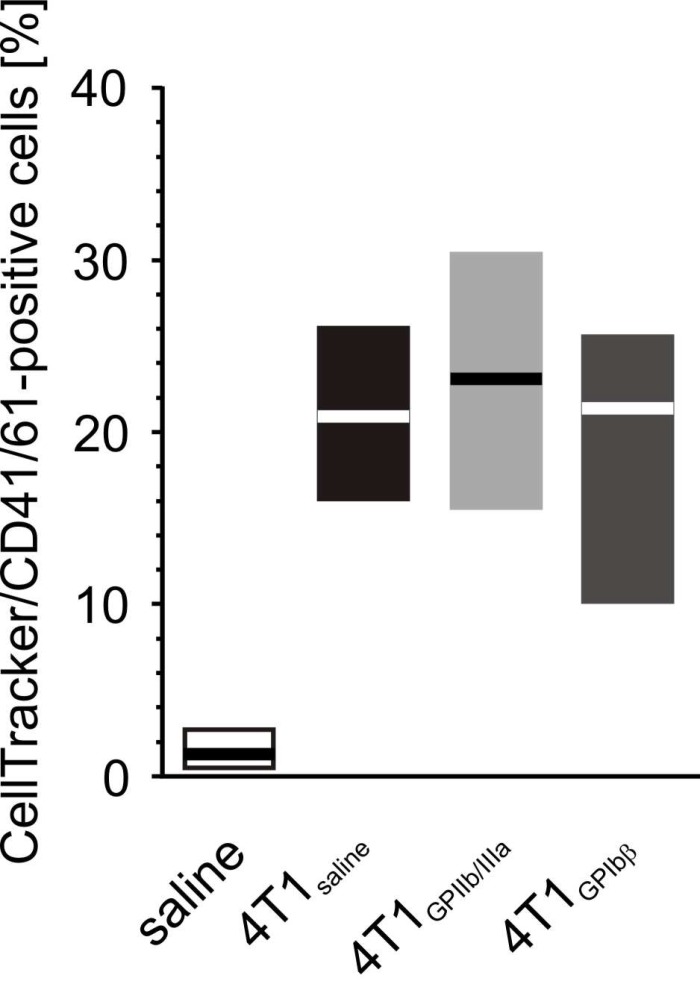
**The effect of the inhibition of GPIIb/IIIa complex and GPIb on the formation of platelet-4T1 aggregates in washed blood samples.** Results are presented as median (horizontal line) and interquartile range (box) (n = 8). Aliquots of washed blood, preincubated with blocking antibodies anti-GPIIb/IIIa (n = 8), anti-GPIbα or saline, were mixed with CellTracker-labeled 4T1 cancer cells or saline. Results are expressed as the percent fraction of CellTracker/CD41/61-positive cells (4T1-platelet aggregates). More experimental details are given in the *Materials and methods* section. The statistical significance of the differences, estimated with the Kruskal-Wallis’ test and the *post hoc* multiple comparisons Conover-Inman test, was: *P*_1,α_ < 0.001, saline < 4T1_saline_ = 4T1_GPIb_ = 4T1_GPIIb/IIIa_.

Increased aggregation of fluorescently-labeled cancer cells with resting platelets was observed (Figure 15), and this aggregation was even more pronounced after platelet stimulation with thrombin. However, the antibodies did not appear to have any significant effects against GPIIb/IIIa or GPIbα: they did not prevent the aggregation between fluorescently-labeled cancer cells and resting platelets.

### Flow cytometric analysis of normoplatelets and platelet-platelet aggregates

The population distributions of normoplatelets were decomposed into two size subpopulations in the control mice and animals with breast cancer; these were referred to as *smaller* and *larger* platelets. Interestingly, the cells appeared to be smaller in 4T1-injected mice than in those treated with saline in both groups of normoplatelets. Moreover, five weeks after the injection of 4T1 cancer cells or saline, the population of smaller normoplatelets decreased in mice with breast cancer, compared to control animals; in contrast, those of the larger normoplatelet fractions increased. The opposite tendency was observed in animals treated with saline. Additionally, four weeks after the injection of 4T1 cells, the population of platelet-platelet aggregates significantly increased in mice developing tumors compared to controls (*P* < 0.01, estimated with one-way ANOVA followed by Tukey’s multiple comparisons test); however, no further changes were observed after five weeks.

## DISCUSSION

Blood platelets can be involved in a number of pathways pivotal for cancer progression and metastasis, including stimulation of tumor angiogenesis and the regulation of circulating tumor cell extravasation to the premetastatic niche in the target organ [33]. The first and the simplest proof of platelet involvement in metastasis revealed in this study is the positive association between the number of blood platelets and the number of metastatic foci, which remains in an agreement with earlier observations [34]. Hence, our findings confirm that platelet number may be a valuable predictor of the metastatic potential of cancer [35]. Since we are aware that platelets are involved in oncogenesis and metastasis, they should be used in clinical practice as diagnostic markers and predictors of metastasis risk, sensitivity to oncostatic therapies and/or target of therapy. The current report focuses on the first of these potential applications.

From a clinical and diagnostic point of view, the most important objective is to obtain a battery of platelet markers suitable for predicting the risk of metastasis in breast cancer. Next to simple morphological markers, such as platelet number, the surface markers of platelet activation, *viz.* an active form of GPIIb/IIIa (fibrinogen receptor) and P-selectin, are suitable candidates, as they are relatively simple to track with flow cytometry and bear some diagnostic potential.

P-selectin mediates platelet adhesion and infiltration into tumors [36]. Resting and agonist-stimulated P-selectin expression is increased on platelets exposed *in vivo* or *in vitro* to cancer cells. Thus, it may well represent the first step of platelet adhesion to the vasculature of the primary tumor and/or to the endothelium of pre-metastatic niche. Our results confirm that the rise of P-selectin expression on blood platelets begins in the early phases of primary tumor growth and this can be a representative platelet predictor of breast cancer progression. Moreover, if platelet P-selectin-targeted is considered to identify metastasis, it can be triggered as early as possible since this platelet marker appears at the early stages of tumor development. However, it remains open to question whether such an early implementation is possible in clinical practice in humans, when breast cancer is often recognized at advanced stage [37].

Our observation that the expression of P-selectin on platelets from mice bearing breast cancer is elevated compared to healthy animals may be explained by the fact that plasma levels of platelet-derived fibrinogen may enhance P-selectin expression under some pathological states [38]. In our study, this hypothesis could be verified by monitoring the level of fibrinogen in plasma from mice injected with saline or 4T1 cells. However, as plasma fibrinogen may be derived not only from platelets, but also from other blood cells or endothelial cells, these findings only indicate the effect of general of plasma fibrinogen level on P-selectin expression in platelets. Interestingly, Coupland and colleagues report that both platelet-derived and endothelial-derived P-selectin appear to contribute to the metastatic process to a similar extent [39].

vWF is widely recognized as a marker of endothelial cell activation; however, its role in tumor-associated platelet activation has not been as thoroughly investigated as that of P-selectin. Nevertheless, it is already known that vWF levels are elevated in the blood of patients with malignant breast cancer and that these levels correlate with tumor progression [40]. The degree of vWF binding by blood platelets is an indicator of the readiness of platelets to interact with the activated endothelium. The results of *in vitro* studies performed with soluble vWF and fibrosarcoma cells indicate that vWF augments cancer cell-induced platelet aggregation by a two-phasic process, in which a close interaction occurs between vWF, platelets and tumor cells, and this is preceded by the adhesion of platelets to vWF [41]. It is possible that cancer cells might somehow prepare the vascular wall for metastasis by increasing vWF expression on the activated endothelium [42], thus facilitating the mentioned adhesion, later followed by aggregation. Interestingly, vWF can react not only with GPIb, but also with GPIIb/IIIa [43]. In the present study it was not possible to evaluate the exact contributions of GPIIb/IIIa or GPIb to vWF binding, since our findings did not distinguish between these two modes of vWF attachment to blood platelets in breast cancer.

However, the fact that GPIb- and GPII/IIIa-blocking antibodies demonstrated no impact on the formation of cancer cell-platelet aggregates *in vitro* suggests that molecules other than GPIb, GPIIb/IIIa, vWF and fibrinogen may be pivotal to their formation. The report by Yokota and colleagues is consistent in some way with our observations, regarding the role of platelet GPIb in tumor metastasis [44]. The authors showed that genetic deletion of platelet GPIb excluded the contributions of this receptors to enhanced platelet-dependent metastasis in hyperthrombotic mice.

Some fluctuations in the expression of GPIIb/IIIa on the ADP-stimulated platelets were identified in the course of five-week breast cancer development, one of which being the increase of activated GPIIb/IIIa complex expression, the receptor for fibrinogen, immediately after 4T1 cell injection, which then fell five weeks later. There are several possible explanations for this trend. Although vWF has affinity for GPIIb/IIIa, in addition to GPIb [45–47], the binding of fibrinogen to GPIIb/IIIa far exceeds that of vWF, at physiological plasma concentrations [48]. Our present findings indicate that GPIb-dependent and GPIbβ-independent binding of vWF to platelets is increased after ADP stimulation, which can probably also occur *via* glycoprotein complex GPIIb/IIIa. vWF and fibrinogen attach to GPIIb/IIIa at different sites of the receptor [49].

The binding of fibrinogen to platelets does not appear to block the access of antibodies to the activated GPIIb/IIIa receptor, as it has been found that JON/A antibodies, *i.e.* the antibodies against the activated GPIIb/IIIa receptors which were also used in our study, does not compete with fibrinogen for the same epitope(s) on GPIIb/IIIa [50]. However, there are no reports whether such competition exists between vWF and antibodies against the activated GPIIb/IIIa.

It cannot be excluded that the reduced binding of anti-activated GPIIb/IIIa antibodies observed in the present study may be attributed to the blockage of the GPIIb/IIIa receptor by higher levels of bound vWF. However, Schober et al. observed that internalization of the activated GPIIb/IIIa receptor was increased in platelets stimulated with ADP, and that the binding of fibrinogen to its receptor had no effect on the internalization of GPIIb/IIIa [51]. This is in line with our present observations, and indicates that: (*i*) the elevated level of fibrinogen released from platelets is bound to the active GPIIb/IIIa complex, and (*ii*) some pool of the activated receptor that is not bound with fibrinogen undergoes internalization, suggesting that the attachment of antibodies against the activated GPIIb/IIIa is reduced.

Recent reports indicate that activated GPIIb/IIIa is absent from procoagulant platelets. Physiological platelet activation leads to the formation of procoagulant platelet subpopulation expressing inactivated GPIIb/IIIa and, surprisingly, high fibrinogen binding levels [52–54]. Thus, it is reasonable to assume that the increased activation of platelets observed at advanced stages of breast cancer development may be associated with elevated fractions of procoagulant platelets, which may demonstrate increased expression of the inactivated GPIIb/IIIa complex. Furthermore, this apparent ‘divergence’ may be also partly explained by the ongoing increased platelet consumption and the phenomenon of GPIIb/IIIa receptor shedding observed during cancer development, probably due to shear stress [55, 56]. However, neither hypothesis mentioned above can account for our present findings, as no significant changes were observed in the total abundance of GPIIb/IIIa in the course of 5-week tumor development compared to healthy mice. In addition, the reduced expression of GPIIb/IIIa observed on platelets after five weeks of breast cancer development may be associated with the increased fractions of platelet-tumor cell aggregates caused by the cross-linking observed between platelet integrins, primarily GPIIb/IIIa, and those expressed on tumor cells, such as α_v_β_3_ [57, 58]. In the present study, the numbers of platelet-cancer cell aggregates increased with almost each week of breast cancer progression, and it cannot be excluded that the GPIIb/IIIa complex plays a considerable role in this process, being one of several platelet receptors involved in the formation of these aggregates with tumor cells.

It is well known that “young”, hyperreactive platelets are usually larger in size than those exhausted due to less or more frequent episodes of activation in the bloodstream. Our findings confirm that these newly-produced, larger normoplatelets are characterized by increased priming and higher sensitivity to subthreshold concentrations of agonists in mice with breast cancer compared to healthy animals. These hyperreactive platelets were also found to aggregate more frequently in mice inoculated with 4T1 cancer cells than in those injected with saline. This observation from our *in vivo* study has been mostly demonstrated in previous *in vitro* studies, in which the aggregation of isolated platelets was measured following treatment with various lines of cancer cells [59]. Zara and colleagues not only demonstrated that cancer cells are capable of inducing platelet aggregation, but also that tumor cell-induced platelet aggregation is not related to the type of the cancer cells, nor to their metastatic potential. Otherwise, it is triggered by platelet activation and secretion driven by the generation of a small amount of thrombin from plasma and it is supported by positive feedback signaling through secreted ADP.

Cancer-induced platelet activation has been demonstrated in several studies, but significantly fewer reports demonstrate the formation of platelet-cancer cell aggregates *in vivo* [60]. The results of the *in vivo* experiments presented herein are in line with those of the *in vitro* part. However, it should be noted that the extent of the altered platelet reactivity or activation monitored in the *in vitro* model was much weaker than that noted in the animals with breast cancer, suggesting that the simplified *in vitro* model lacks several of the crucial factors present in a cancer-bearing host organism. The *in vivo* model includes numerous factors, including the activity of the vascular endothelium, which probably are not changed within a short time after the injection of cancer cells (time point t_0_) and this physiological activity inhibits the possible activation of platelets caused by the presence of 4T1 cells. However, after two weeks from the administration of cancer cells, when breast tumors are visible, the overall functioning of the vascular endothelium, and hence its antiplatelet activity, could be impaired [61]. This may be manifested by increased platelet priming, which was observed in our study two weeks after tumor induction. The *in vitro* approach offers a more isolated environment, where the only elements interacting with each other are blood cells (including platelets) and cancer cells, and as such, the effect of 4T1 cells on platelets is not affected by factors that can be present *in vivo*. Of course, this is just one of the hypotheses explaining the observed dissimilarities between the *in vitro* and *ex vivo/in vivo* models.

Therefore, our *in vitro* results failed to confirm the above described tendency: despite being significantly higher than control values, the activation and reactivity of blood platelets exposed to cancer cells under *in vitro* conditions were found to be rather weak, and did not reflect the high number of heteroaggregates of blood platelets and cancer cells present in mice inoculated with 4T1 cells. *In vitro* formation of platelet-4T1 cell aggregates was found to be increased after platelet stimulation with thrombin. Therefore, under *in vitro* conditions, stimulated platelets appear to interact with 4T1 cancer cells more readily than non-stimulated platelets. It should be mentioned that thrombin-driven interactions between platelets and cancer cells is only one of the protease-activated receptor (PAR)-dependent mechanisms known to exist. Another one, described by Yokota et al., identified PAR1 signaling on both tumor and host cells as a contributor to metastasis in hyperthrombotic mice; the authors indicate that procoagulant activity of tissue factor (TF) plays a crucial role in successful metastasis by improving intravascular tumor cell survival through fibrin formation, platelet activation and platelet-dependent protection from natural killer cell attack [44]. High levels of TF expression have been observed on 4T1 cancer cells in a murine breast cancer model [62, 63]. Interestingly, Gomes and colleagues proved that not only cancer cells, but also cancer cell-derived extracellular vesicles (EVs), with a high surface expression of TF, can induce TF-dependent plasma clotting and platelet aggregation by means of thrombin generation, in addition to the TF-independent mechanism of platelet activation expressed by these vesicles [64]. Coupland and colleagues, in turn, showed that cell lines with negative surface expression of TF are unable to initiate coagulation via factor VII binding or activation, with subsequent thrombin generation [39]. Hence, TF may act according to a time-dependent manner of platelet activation and tumor metastasis, as observed in our present study: cancer cells, the number of which increases with tumor development, generate thrombin through surface TF and thus stimulate platelets which, in turn, facilitate the migration of cancer cells.

We propose that the *in vitro* surface membrane expression of proteins commonly considered the hallmarks of platelet activation and reactivity is indeed increased, but it does not achieve high values. Such elevated, but still relatively low, platelet activation is sufficient to induce interaction between blood platelet and cancer cells, and thus trigger platelet-dependent metastatic spread. However, platelet activation cannot propagate infinitely and cannot be increased further, as it will induce the formation of hemostatic plugs by platelet aggregates, thus preventing blood flow. This, in turn, would immobilize blood platelets with attached cancer cells and would obviously terminate metastasis. Therefore, the presence of low platelet activation and high numbers of platelet-cancer cell heteroaggregates has reasonable biological significance and is necessary to harness blood platelets as efficient ‘Trojan horses’ to spread cancer cells.

Our findings indicate strong relationships between the parameters of platelet activation and the number of metastases in the lungs. These results may support the theory that platelets can facilitate the process of metastasis following activation by their interactions with cancer cells. The fact that an elevated number of platelets and leukocytes was observed at advanced stages of tumor development is consistent with the phenomenon of increased extramedullary hematopoiesis in the liver of mice with breast cancer. Moreover, it seems that this high platelet count observed in the late weeks of tumor development consisted of larger and hence, of newly-synthesized and more reactive platelets. The decreasing fraction of lymphocytes observed in the present study during the progression of breast cancer may be explained by the fact that peripheral blood lymphocyte count is believed to influence survival in breast cancer [65]. Moreover, studies have attributed the presence of a reduced lymphocyte count to the reduction of the lymphocyte fraction; the latter is caused by the increase in the number of myeloid tumor suppressor cells characteristic of the development of cancer [66]. Similarly, our study based on a mouse model of breast cancer found a low number of lymphocytes and reduced platelet count to be related with decreasing survival at the advanced stages of tumor progression, *i.e.* after five weeks from the inoculation of mice with 4T1 cancer cells. Other reports indicate a loss of the lymphocyte fraction in peripheral blood, which may be caused by the accumulation of so-called tumor-infiltrating lymphocytes (TIL) around the site of primary tumor proliferation [67, 68].

In addition, our findings indicate the presence of strong associations between the parameters of platelet activation, blood cell counts and variables describing breast cancer metastases in lungs, as well as those characterizing the extramedullary hematopoiesis foci present in liver. So far, no causes of this type of hematopoiesis have been identified in the development of cancer. Extramedullary hematopoiesis is most frequently seen in hematological disorders, where massive infiltration of bone marrow leads to stem cell migration [69]. Moreover, it is also identified as a compensatory phenomenon occurring in states of insufficient bone marrow function and as a secondary or accessory event to other disorders, including cancer [70], in certain cases developing independently, lacking an apparent trigger and without clinical or diagnostic implications [71, 72]. In cancer, extramedullary hematopoiesis appears quite rare: the incidence among cancer patients with metastatic disease is less than 10% [73]. The most commonly involved solid tumors are lungs, breast and prostate, with a late onset in advanced stages, when the function of the bone marrow is severely disturbed [74].

## CONCLUSIONS

To briefly summarize our present findings, we have found that platelet activation and reactivity increase during the growth of the primary breast tumor and they are strongly associated with metastasis and the formation of secondary tumors in lungs. This process is augmented by numerous newly-synthesized platelets produced, among others, in the liver in the course of extramedullary haematopoiesis. Our results support the notion that blood platelets play a key role in metastasis, and therefore, that antiplatelet therapy, started as early as possible, could reduce the risk of secondary tumor development.

## MATERIALS AND METHOD

### Chemicals

Anesthetics: sedazin (20 mg/ml xylazine) and ketamine (100 mg/ml ketamine hydrochloride) were obtained from Biowet (Biowet, Pulawy, Poland). Low molecular weight heparin (LMWH) was from Sanofi Aventis (Paris, France). FITC- or PE-conjugated rat anti-CD41/61, PE-conjugated rat anti-CD62P (rat anti-P-selectin), PE-conjugated JON/A antibodies (rat anti-active complex GPIIb/IIIa), FITC-conjugated rat anti vWF and rat anti-fibrinogen antibodies were purchased from Emfret Analytics (Eibelstadt, Germany).

Platelet surface receptors were blocked using the Leo.H4 clone of monoclonal rat anti-mouse GPIIb/IIIa antibodies (at the dose of 20 μg/ml), which blocks the binding of fibrinogen to the (activated) receptor, and the Xia.B2 clone of monoclonal rat anti-mouse GPIbα antibodies (at a dose of 20 μg/ml), which blocks the vWF binding site on the GPIbα. Both antibodies were used at their saturating concentrations, as indicated in the manufacturer’s instructions (Emfret Analytics, Eibelstadt, Germany). The information on the blocking abilities of these clones has been provided in the manufacturer’s instruction while these properties have been confirmed in previous studies [75–83]. These antibodies do not induce platelet activation after binding to appropriate receptor.

PerCP-conjugated rat anti-CD24 and APC-conjugated rat anti-CD44 antibodies were obtained from Becton Dickinson (San Jose, CA, USA). Thrombin from human plasma was purchased from Chronolog Co. (Havertown, PA, USA). Reagents for the preparation of histopathological specimens were from Leica Biosystems (Wetzlar, Germany). Inorganic salts and all other chemicals were purchased from Sigma-Aldrich (St. Louis, MO, USA), unless otherwise stated. Water used for solution preparation and glassware washing was passed through an Easy Pure UF water purification unit (Thermolyne Barnstead, IA, USA).

### Cell culture

The mouse mammary adenocarcinoma 4T1 cells and breast epithelium non-cancer EpH4-Ev cells were obtained from the American Type Culture Collection (ATCC, USA). The 4T1 cell line was maintained in the Institute of Immunology and Experimental Therapy, Wroclaw, Poland. Cells were cultured as described elsewhere [84].

### Murine model of metastatic breast cancer

All experiments were performed in accordance with the guidelines formulated by the European Community for the Use of Experimental Animals (L358-86/609/EEC) and the Guide for the Care and Use of Laboratory Animals published by the US National Institute of Health (NIH Publication No. 85–23, revised 1985). All procedures used in these experiments were approved by Local Ethics Committee on Animal Experiments at the Medical University in Lodz (approval number: 68/ŁB05/2015).

Balb/c female mice were purchased from the Center of Experimental Medicine, Medical University of Bialystok, Poland. The mouse model of breast cancer was developed by the orthotopic injection of viable 4T1 cancer metastatic cells (1 × 10^4^ cells in the Hank’s Balanced Salt Solution) into mammary fat pads in eight-week-old Balb/c female mice (average body weight = 21.5 g ± 1.3). Animals injected with natrium chloride (0.9%) served as controls. All injections were performed on the same day. Allocation to experimental groups was based on simple randomization. During the experiments, the animals were housed under standard conditions and constant veterinary supervision, with free access to water and standard chow for rodents (Altromin Maintenance Diet). Blood was obtained immediately (two hours, t_0_) after cancer cell injection and after two (t_2_), three (t_3_), four (t_4_) and five (t_5_) weeks of cancer development (n=8 for each time interval) as described previously [76]. Blood count was performed directly after blood collection using a blood counter (abc Vet, Horiba).

### Blood sampling

During the experiments, the animals were housed in an isolated room with a 12-hour light-dark cycle in standard plastic cages (transparent) filled with mulch typically used in rodent breeding, and were given free access to water and standard chow for rodents (Altromin Maintenance Diet). The animals were under constant veterinary supervision: routine veterinary inspections assessing the overall welfare of the animals were carried out daily. The mice were anaesthetized (at the laboratory) with an intramuscular injection of ketamine (100 mg/kg b.w.) and xylazine (23.32 mg/kg b.w., anaesthesia suitable for platelet measurements). Blood was terminally collected from the inferior aorta on 10 U/ml LMWH in TBS buffer (20 mmol/l Tris-HCl, 137 mmol/l NaCl, pH 7.3).

### Evaluation of platelet activation and reactivity and estimation of platelet-cancer cell aggregates in peripheral blood using flow cytometry

Circulating platelet activation, and platelet reactivity in response to ADP (5 or 20 μM) and thrombin (0.025 or 0.25 U/ml) were evaluated on the basis of the measured expressions of specific surface membrane antigens: CD62P (P-selectin), activated GPIIb/IIIa complex, binding of endogenous vWF and binding of endogenous fibrinogen to the platelet surface. Flow cytometric protocol was used as described previously [85]. Results were presented as the percent fraction for P-selectin-, activated GPIIb/IIIa-, vWF- or Fg-positive platelets.

Platelet-cancer cell aggregates were identified on the basis of the expression of CD24 and CD44 within the population of CD41/61-positive objects (platelets) and not *vice versa*, since the number of cancer cells in bloodstream was expected to be certainly smaller than the number of circulating platelets, even at the advanced stages of tumor development. CD24 and CD44 antigens were chosen as they are found to be the surface markers of many human and mouse cancer cells, including breast cancer [86, 87]. CD24 is found to be expressed on more differentiated cancer cells, whereas CD44 is usually present on stem-like cells [88]. Thus, both markers of two subpopulations of breast cancer cells were identified in our study, to avoid accidentally losing one of the subpopulations. The 4T1 cancer cells used in this study are well characterized by high expression of CD24 and CD44 [89].

### Size-based evaluation of changes in normoplatelets and platelet aggregates during five-week cancer development

The groups of newly-produced (large, “fresh”) or exhausted (small, “old”) platelets in the population of single platelets, as well as in the population of single and aggregated platelets (CD41/61-positive cells) in resting, non-stimulated blood were differentiated on the basis of platelet size, as previously described [75].

### Activation of blood platelets by cancer cells *in vitro*

Blood obtained from inferior aorta of female Balb/c (*n*= 8) was diluted 25-fold in Tyrode’s buffer and incubated with 4T1 cell suspension (1 x 10^5^ cells/ml) or with saline (control) for 15 min at 37°C. Platelet activation was evaluated by the procedure described above. The aggregates of platelets with cancer cells were measured after the incubation of whole blood samples with 4T1 cells (15 min, 37°C), pre-labeled with CellTracker (30 min, 37°C): the cells were detached using sodium citrate instead of trypsin, as our previous experiments indicate that trypsin artefactually activates platelets. The ability of anti-GPIIb/IIIa and anti-GPIb monoclonal antibodies to inhibit interactions between cancer cells and blood platelets was evaluated by incubating the antibodies with whole blood samples (15 min, room temperature), before adding CellTracker-labeled 4T1 cells. The formation of aggregates was enhanced by thrombin stimulation of platelets (0.25 U/ml, 15 min, room temperature). The percentage fraction of CellTracker–positive cells within the CD41/61-positive platelets was estimated.

### Histopathological evaluation of metastasis and extramedullary hematopoiesis in a mouse model of breast cancer

Breast cancer metastasis in the lungs and extramedullary hematopoiesis in the liver and spleen were investigated by routine histopathological examination after fixation in 10% buffered formalin for at least 72 hours. The histological appearance of the tissue was examined by light microscopy and images were taken with the use of inverted microscope with a standard color camera.

The lung metastases were ascertained in three following sections under magnifications of 40X, 100X and 400X. The detailed size of each section was measured, the metastases were quantified and the largest dimension of metastatic foci for each section was measured.

The extramedullary hematopoiesis foci in liver were identified in three subsequent sections under magnifications of 40X, 100X and 400X. They were distinguished from inflammatory foci by the presence of bone marrow progenitor cells including nucleated erythrocytes, immature granulocytes, or megakaryocytes, in the absence of associated hepatocellular necrosis [90, 91]. The extramedullary hematopoiesis foci were quantified using a semiquantitative scoring method combining a 4-point score representing the number of extramedullary hematopoietic foci (0 points: no foci and 4 points: highest number of foci) with another 4-point score quantifying the extent of extramedullary hematopoiesis (0 point: no foci and 4 points: very high extent of extramedullary hematopoiesis).

### Immunohistochemistry detection of extramedullary hematopoietic markers in liver

To confirm the presence of extramedullary hematopoietic foci in liver, routine immunohistochemical staining was performed with the use of the following antibodies against hematopoietic markers: primary rabbit anti-mouse antibodies for CD117 (erythroid marker) (clone A4502), FVIII (hematopoietic marker for megakaryocyte) (clone IR527) and MPO (granulopoietic marker) (IR/IS511). The images were taken using an AxioObserver D1 inverted fluorescent microscope and AxioCam HRm monochromatic digital camera (Carl Zeiss, Oberkochen, Germany). Image analysis was performed semi-automatically using Axio Vision 4.8 software (Carl Zeiss, Oberkochen, Germany).

### Statistical analysis

Data are presented as mean + SD or median (*Me*) and interquartile range (IRQ, lower [25%] quartile to upper [75%] quartile, *Me*; LQ-UQ), depending on data scale and distribution. The normality of the data distributions was verified with the Shapiro-Wilk test, variance homogeneity was tested with Levene’s test and sphericity was verified with Mauchly’s test. The Mann-Whitney *U*-test was employed to evaluate the significance of differences between two independent groups of discrete variables or variables departing from normality. Otherwise, Student’s *t*-test for independent samples was used for comparing two groups. One-way or two-way block ANOVA (with relevant *post hoc* Tukey’s multiple comparisons test) was used to compare multiple data sets, while the Kruskal-Wallis test followed by the *post hoc* all-pairwise comparisons Conover-Inman test was used for data not satisfying the criterion of normality and/or variance homogeneity. The Spearman's rank correlation coefficient was used to calculate correlation coefficients between selected variables: (a) platelet and leukocyte counts, fractions of lymphocytes and granulocytes, (b) variables describing breast cancer metastases in lungs and the extramedullary hematopoiesis foci in the liver.

Canonical analysis was employed to determine which of the studied variables contributed the most to the significant association between three sets of variables: (a) variables describing blood platelet activation and reactivity, (b) variables describing breast cancer metastases in lungs, and (c) variables characterizing the extramedullary hematopoiesis foci in liver. The aim was to build up a canonical (common) variable for each examined set of variables and to estimate the associations between different canonical variables describing different sets of parameters, thus identifying the variables forming the strongest associations between blood platelet functioning and cancer metastases or extramedullary hematopoiesis.

Due to the relatively small sample sizes and the low statistical power of the estimated inferences in some calculations, the resampling bootstrap technique (1000-10,000 iterations) was used to determine the likelihood of obtaining the revealed differences due to pure chance; in such circumstances, we refer to the bootstrap-boosted test statistics (10,000 iterations) instead of the classical approach. For some variables, the overall population of data was decomposed into two exponentially-modified Gaussian partial distributions (subpopulations) with the use of the Data Mining-assisted generalized cluster analysis employing the EM (expectation-maximization) algorithm. For each experiment, sample size was estimated based on the expected standardized effect, the accepted significance (corrected for multiple comparisons) and the accepted statistical power, with regard to the employed experimental design. Statistical analysis was performed using Statistica v. 12.5, Resampling Stats Add-in for Excel v. 4 and StudSize3.
